# Enhancing Chimeric Fragmentation Spectra Deconvolution Using Direct Infusion–Tandem Mass Spectrometry Across High‐Resolution Mass Spectrometric Platforms

**DOI:** 10.1002/rcm.10170

**Published:** 2025-11-16

**Authors:** Arina Ivanova, Wei Tang, Carsten Simon, Kai Dührkop, Sebastian Böcker, Gerd Gleixner

**Affiliations:** ^1^ Department of Biogeochemical Processes Max Planck Institute for Biogeochemistry Jena Germany; ^2^ Chair for Bioinformatics Friedrich Schiller University Jena Jena Germany

## Abstract

**Rationale:**

Direct infusion mass spectrometry (DI‐MS) is a rapid analytical technique widely used in omics research and other fields. However, the complexity of DI‐MS spectra frequently leads to co‐fragmentation of analytes with similar *m/z*, resulting in chimeric fragmentation spectra that complicate compound identification. A DI‐based tandem mass spectrometric method (DI‐MS2), which modulates the intensity of precursors and fragments by the stepwise movement of the quadrupole isolation window, has been shown to successfully deconvolute chimeric fragmentation spectra. Yet, its applicability to different instruments and optimisation has not been evaluated.

**Method:**

We evaluate the performance of DI‐MS2 on two high‐resolution instruments: a linear ion trap‐Orbitrap (LIT‐Orbitrap) and a quadrupole‐Orbitrap (Q‐Orbitrap). We examined the impact of six instrumental settings, including mass resolving power, isolation window width, step size between MS2 scans, number of microscans, collision energy and automatic gain control (AGC) target, on the analysis of isobaric mixtures with varying *m/z* differences.

**Results:**

The LIT‐Orbitrap consistently achieved high‐quality chimeric spectra deconvolution with an average similarity score of 0.98 despite unexpected intensity modulation patterns. The Q‐Orbitrap provided four times faster measurements but showed more variable results: It achieved a similarity score of 0.96 for isobars with a *m/z* difference larger than 0.02, but only 0.56 for *m/z* differences of 0.006.

**Conclusions:**

These findings indicate that the DI‐MS2 is a robust and flexible method applicable across different MS platforms, though the Q‐Orbitrap may be less suited for highly complex samples with multiple peaks per nominal mass. This highlights the potential of the DI‐MS2 for structural elucidation of complex biological mixtures. Additionally, we provide initial setting optimisation guidelines to improve spectra deconvolution and measurement speed.

## 1 Introduction

Direct infusion mass spectrometry (DI‐MS) is a fast [1, 2] analytical tool used in biomarker discovery [3, 4], environmental monitoring [5], metabolomics [6, 7] and proteomics [8, 9]. In DI‐MS, the sample is not subjected to additional separation like chromatography or capillary electrophoresis, which speeds up the analysis but results in thousands of molecules being ionised and detected simultaneously. Such spectral complexity of DI‐MS data leads to isobaric interferences, where multiple ions are present at the same nominal *m/z* value. Usually, the range of *m/z* values that are isolated for further fragmentation is relatively wide: Often, an isolation window width between 1 and 2 *m/z* is used [10, 11, 12, 13]. Due to the physics of imperfect band‐pass filtering, commercial instruments impose a lower bound on the values a user may enter as isolation range, such as 0.1 or 0.4 *m/z* for different Orbitrap instruments, which leads to isobaric interferences in complex samples [14, 15]. Smaller isolation windows inevitably result in a loss of sensitivity, as fewer ions are allowed to pass through and fragment. As a consequence, ions with close *m/z* values co‐fragment and produce chimeric MS2 spectra containing multiple precursors and their fragments. Such chimeric fragmentation spectra can massively hinder compound identification [16, 17].

Numerous strategies have been suggested to tackle the analysis of chimeric spectra in DI‐MS2 analyses. One way to detangle chimeric MS2 spectra into individual fragmentation spectra is by matching the mass differences between precursors and fragments [18]. For each possible fragment‐precursor pair, a mass difference is calculated, and the fragment is only assigned to the precursor if the mass difference matches the mass differences found in MS2 databases or in‐house libraries. Although this approach has been shown to improve the assignment of molecular formulae and chemical classes, its effectiveness relies on the list of allowed mass differences. Consequently, it may be biased by the database from which the differences were sourced, potentially overlooking all mass differences that are underrepresented or missing in databases.

**FIGURE 1:**
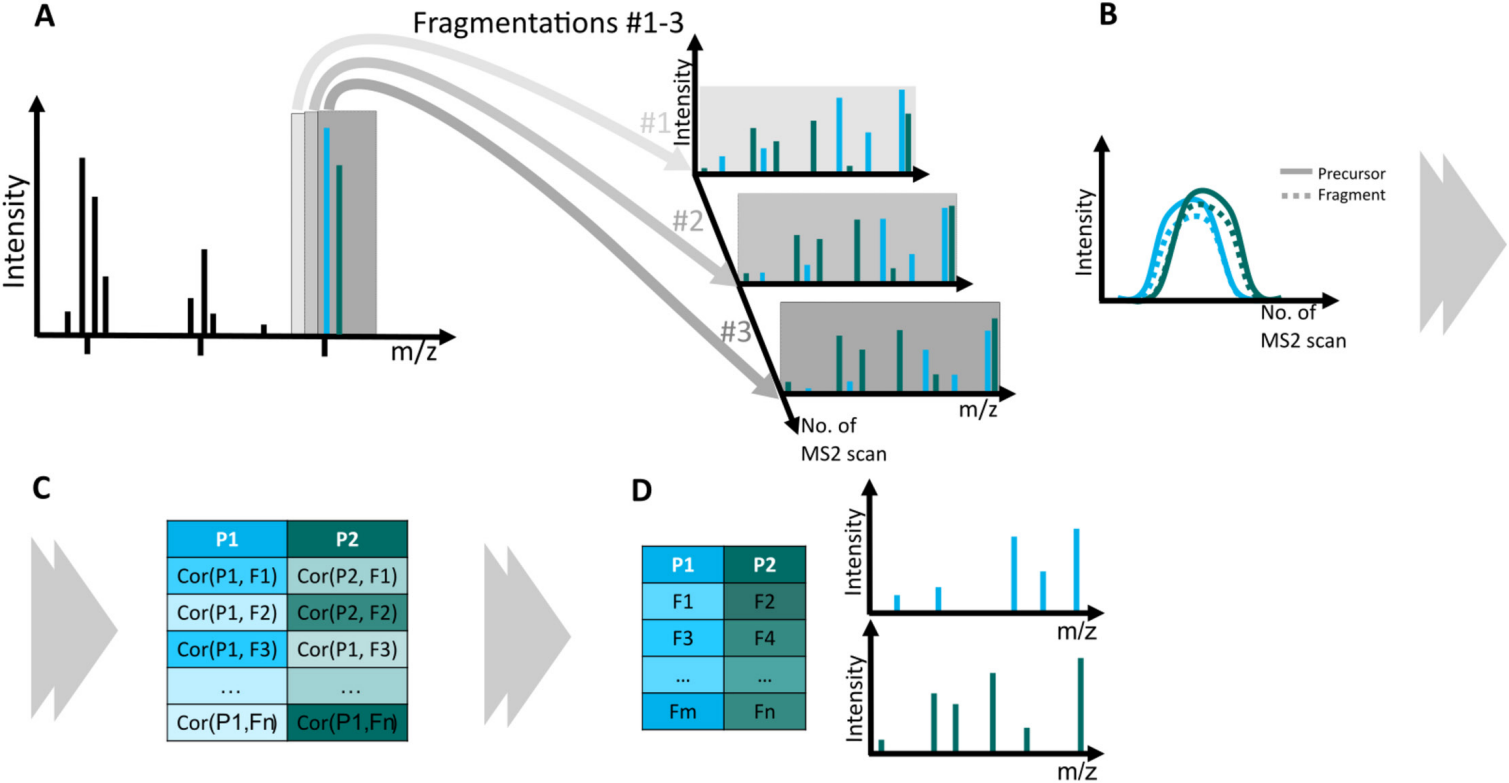
The principle of the DI‐MS2 method: (A) Multiple MS2 scans are recorded over the *m/z* range of interest, with the centre of the isolation window being shifted stepwise for each MS2 scan. The transmission of ions with *m/z* close to the centre of the isolation window is better, resulting in higher intensity in an MS2 spectrum. In such manner, the precursor ion's intensity—and, consequently, the intensity of its fragments—is modulated across MS2 spectra depending on precursor's position within each isolation window. (B) If the isolation window is being shifted from lower to higher *m/z* values, the signals of precursor with lower *m/z* (here, P1) and its fragments appear first in MS2 spectra, followed by precursor with heavier *m/z* (here, P2). The intensity modulation by shifting isolation window creates two distinct intensity profiles for precursors P1 and P2. (C) Based on collected MS2 scans, a matrix of correlation coefficients between precursors (P1, P2) and all possible fragments (F) Cor (*P*_*i*_, *F*_*j*_) is calculated. The differences in modulated intensities of precursors P1 with its fragments and P2 with its fragments result in correlation coefficients being higher for correct combinations of a precursor and a fragment, allowing for (D) the reconstruction of individual fragmentation spectra. In this study, reconstructed fragmentation spectra from isobar mixtures were compared with reference spectra obtained from the MS2 analysis of individual isobaric compounds.

An alternative approach reconstructs individual fragmentation spectra from chimeric DI‐MS2 spectra without relying on database information, using a different data acquisition technique [19]. This method is conceptually related to SONAR [20] and scanning SWATH [21] data acquisition methods, which were developed for liquid chromatography–mass spectrometry and acquire fragmentation spectra by shifting a relatively wide quadrupole isolation window (10–24 *m/z*) across the mass range of interest to fragment all co‐eluting compounds. However, in the DI‐MS2 adaptation the quadrupole isolation window is more narrow and is shifted in small increments across the smaller targeted *m/z* range (Figure 1). Due to the imperfection of the band‐pass quadrupole filter, ions with *m/z* ratios close to the centre of the isolation window are transmitted more efficiently, resulting in higher intensity in the MS2 spectrum. As the isolation centre shifts, the intensity of the precursor ion—and consequently its fragments—is *modulated* based on its position within the isolation window. This stepwise isolation of *m/z* values from smaller to higher (from left to right in mass spectrum) causes different isobars to appear in MS2 spectra at different times, depending on their position within the isolation window at each step (Figure 1B). The deconvolution of chimeric spectra then relies on the differences in modulated intensity between the isobars. Importantly, this process is independent of the instrument's resolution and mass accuracy; it is solely due to the imperfection of the band‐pass filter that is selecting ions for fragmentation. Although this DI‐MS2 method has successfully deconvoluted chimeric spectra, it has only been applied to a quadrupole‐based mass spectrometer with a single set of settings focusing on compounds with *m/z* below 200 and relatively simple fragmentation patterns. It remains unclear whether chimeric spectra of any isobaric mixture, regardless of their *m/z* values and complexity of fragmentation spectra, can be deconvoluted, or whether isobars with very close *m/z* values present challenges for the method. If this DI‐MS2 method could be adapted to various mass spectrometric platforms and optimised for rapid and reliable measurements, it would provide new, complementary insights into the composition of complex biological samples [16].

It is well known that the type of MS platform significantly affects the quantity and quality of produced MS2 spectra due to differences in fragmentation mechanisms and spectra acquisition speed [22]. The variations in instrumental setup may be even more influential for any method that relies on the movement of the isolation window by a low‐resolution mass analyser. Moreover, the analysis time and quality of MS2 spectra, which are crucial for high‐throughput analysis and compound identification, strongly depend on various instrumental settings. High mass resolving power enhances the separation of overlapping peaks in complex MS spectra, thereby improving mass accuracy, though it results in a longer measurement cycle [23, 24]. The number of microscans per scan [24, 25] and automatic gain control target value (AGC) [25, 26] influence both sensitivity and measurement time. The type of fragmentation and the applied collision energy influence the quality of the produced fragmentation spectra [27, 28]. The width of the isolation window is changed to improve sensitivity or reduce co‐fragmentation and contamination of fragmentation spectra [12]. We expect that the step size between consecutive scans, a parameter innate to the described DI‐MS2 method, is important for its performance, as it defines the modulation of intensities. Thus, broadening the applicability of this promising DI‐MS2 technique requires testing across various mass spectrometric setups and instrumental settings.

To explore the applicability of the DI‐MS2 method across different setups, we used two high‐resolution mass spectrometers with different low‐resolution mass analysers, that is, a linear ion trap–Orbitrap instrument (LIT‐Orbitrap) and a quadrupole–Orbitrap instrument (Q‐Orbitrap). We created a set of 17 different instrumental methods varying six key settings: mass resolving power, isolation window width, step size between MS2 scans, number of microscans, collision energy, and automatic gain control target. We hypothesised that the method can be effectively used regardless of the high‐resolution mass spectrometers set up, yielding consistent results. Among these parameters, the step size between consecutive MS2 events is expected to be particularly important, as it influences the intensity modulation and potential for chimeric spectra deconvolution. To assess the impact of these instrumental settings, we analysed three mixtures of isobaric compounds with molecular weights between 180 and 342 Da and differences between molecular ion *m/z* values ranging from 0.006 to 0.048. We expect that isobars with smaller *m/z* differences will exhibit more similar intensity modulation, making the deconvolution of their chimeric MS2 spectra into individual MS2 spectra more challenging.

## 2 Experimental Section

### 2.1 Chemicals

**FIGURE 2:**
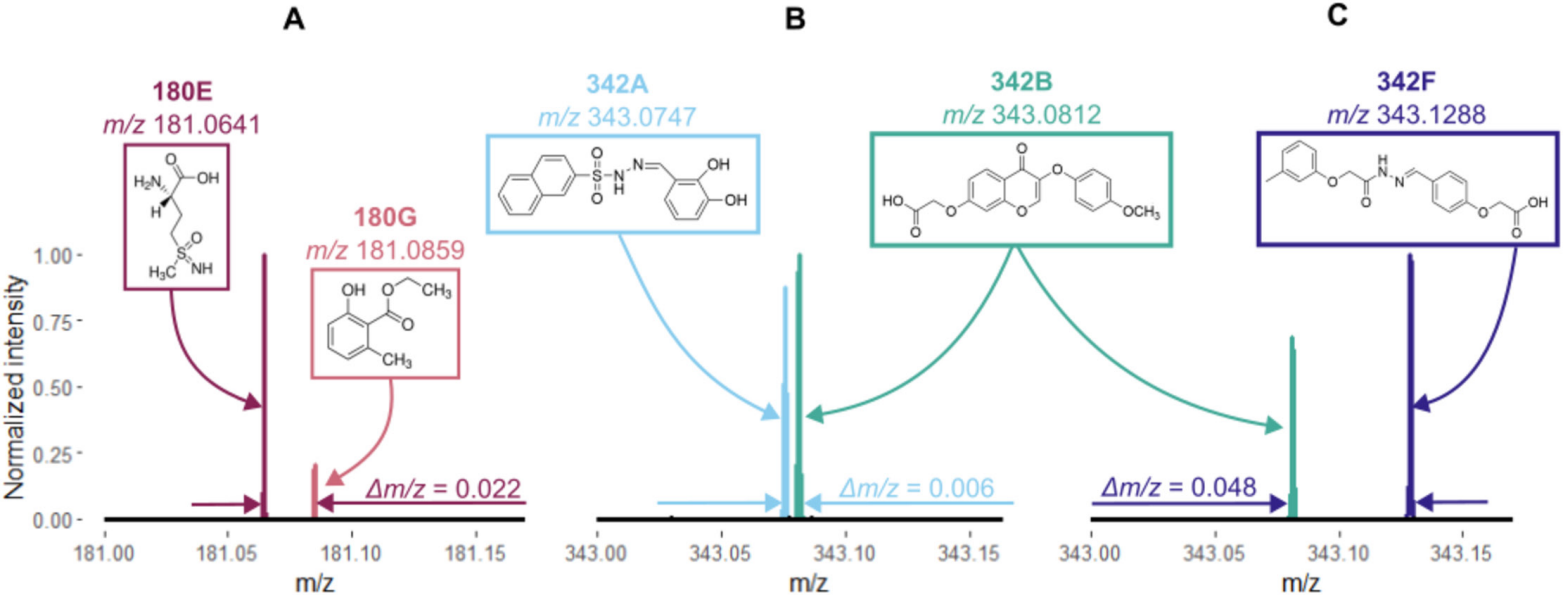
The sections of MS1 spectra of isobaric mixtures used in the method testing. Additionally, molecular structures, *m/z* values for [M + H]^+^ ions and difference between [M + H]^+^ *m/z* values of isobaric compounds *Δm/z* are given. Spectra were recorded at mass resolution of 480 000 on LIT‐Orbitrap: (A) Mixture 180E + 180G (180E is 2‐amino‐4‐(methylsulfonimidoyl)butanoic acid; 180G is ethyl 2‐hydroxy‐6‐methylbenzoate); (B) mixture 342A + 342B (342A is *N*′‐[(Z)‐(2,3‐dihydroxyphenyl)methylidene]naphthalene‐2‐sulfonohydrazide; 342B is {[3‐(4‐Methoxyphenoxy)‐4‐oxo‐4H‐chromen‐7‐yl]oxy}acetic acid); (C) mixture 342B + 342F (342F is 2‐[4‐[(E)‐[[2‐(3‐methylphenoxy)acetyl]hydrazinylidene]methyl]phenoxy]acetic acid).

We used three mixtures of isobaric compounds to evaluate isobaric interferences. Each mixture had two isobaric compounds with a molecular weight of 180 or 342 Da and varied difference between *m/z* values of [M + H]^+^ ions (Figure 2 and Table S1): The first mixture 180E + 180G had a nominal mass of 180 Da and had a *m/z* difference of 0.022. Compound 180E was 2‐amino‐4‐(methylsulfonimidoyl)butanoic acid, purchased at ≥ 95% purity from Sigma‐Aldrich, United States; compound 180G was ethyl 2‐hydroxy‐6‐methylbenzoate, purchased at ≥ 95% purity from Sigma‐Aldrich, United States. The second mixture 342A + 342B had a nominal mass of 342 Da and *m/z* difference of 0.006. Compound 342A was *N*′‐[(Z)‐(2,3‐dihydroxyphenyl)methylidene]naphthalene‐2‐sulfonohydrazide, purchased at ≥ 90% purity from Vitas M Chemical Limited, China. Compound 342B was {[3‐(4‐methoxyphenoxy)‐4‐oxo‐4H‐chromen‐7‐yl]oxy}acetic acid, purchased at ≥ 95% purity from Sigma‐Aldrich, United States. The third mixture 342B + 342F also had a nominal mass of 342 but the *m/z* difference of 0.048 was larger than in the second mixture. Compound 342F was 2‐[4‐[(E)‐[[2‐(3‐methylphenoxy)acetyl]hydrazinylidene]methyl]phenoxy]acetic acid, purchased at ≥ 95% purity from Sigma‐Aldrich, United States. All three isobaric mixtures can be separated in MS1 but would co‐fragment in the MS2 experiment as the minimal *m/z* isolation range of Q‐Orbitrap (0.4) and LIT‐Orbitrap (0.1) is larger than the *m/z* differences of the mixtures.

The solvents used were UHPLC–MS grade MeOH (HiPerSolv CHROMANORM, VWR, Germany), LC–MS grade DMSO (Pierce, Thermo Scientific), ultrapure water (Milli‐Q IQ 7000, Merck) and LC–MS grade HCOOH (VWR Chemicals). The stock solutions of used compounds were prepared at concentration 1000 ppm in MeOH (180E, 180G, 342B) or DMSO (342A, 342F) depending on the solubility. The mixtures of compounds and solutions of pure individual compounds were prepared by diluting stock solutions in MeOH:H_2_O 50:50 (v./v.) solvent with addition of 0.1% (v.%) HCOOH to improve ionisation in positive mode. The concentrations of individual solutions for measurements of reference spectra were 5 ppm for compounds 180E, 180G, 342A, and 342B and 10 ppm for the compound 342F. The concentrations of compounds in mixtures were the following: Mixture 180E + 180G had concentration 5 ppm of 180E and 5 ppm of 180G; mixture 342A + 342B had concentration 5 ppm of 342A and 0.5 ppm of 342B; mixture 342B + 342F had 1 ppm of 342B and 5 ppm of 342F. The concentrations of mixtures were chosen in such way that the signal intensities of isobars in MS1 were roughly equal. As a blank, the same solvent with addition of 1% (v.%) DMSO was used.

### 2.2 Measurements

All measurements were performed using two high‐resolution mass spectrometers: a linear ion trap–Orbitrap instrument Orbitrap Elite (LIT‐Orbitrap) and a quadrupole‐orbitrap instrument Orbitrap Exploris 120 (Q‐Orbitrap; both from Thermo Fisher Scientific). All data were collected in the profile mode via direct infusion with positive electrospray ionisation using source settings optimised to produce a stable electrospray for each instrument (Table S2). In all tandem mass spectrometric measurements (MS2), we used higher energy collisional dissociation (HCD) fragmentation and normalised collision energy (NCE).

The reference spectra of pure compounds were collected with a mass resolving power of 120 000 (as given by the manufacturer at *m/z* 400 for Orbitrap Elite and at *m/z* 200 for Orbitrap Exploris 120) and a 1 *m/z* isolation window width using the same collision energies that were used for the analysis of isobaric mixtures, namely stepped NCE 35‐45‐55 and separate NCEs 35, 50, and 65. Additionally, blank fragmentation spectra were collected for the solvent using the same settings but with a 2 *m/z* isolation window width for blank subtraction.

The analysis of isobaric mixtures uses a data‐independent acquisition technique that enables chimeric spectra deconvolution through intensity modulation by stepwise shifting of the MS2 isolation window [19]. We established a default method as reference (Table 1), which included 81 fragmentation events. These events began with fragmentation at *m/z* value (*x* − 0.7), where *x* represents the nominal mass of the isobars. Each subsequent fragmentation occurred in 0.02 *m/*z increments, continuing until *m/z* value (*x* + 0.9), covering the range of *m/z* values from (*x* − 0.7) to (*x* + 0.9) in one measurement cycle. To assess the impact of different settings on analysis time and the quality of chimeric spectra deconvolution, we altered one setting at a time in the default method. For a wider isolation window of *m/z* 2, the fragmentation started at *m/z* (*x* − 1.7) and ended at *m/z* (*x* + 1.9) resulting in 137 fragmentation events that captured the ‘edges’ of modulated intensities. For a narrower isolation window of *m/z* 0.4, the fragmentation started at *m/z* (*x* − 0.3) and ended at *m/z* (*x* + 0.4) creating 51 fragmentation events. In total, 17 measurement methods were used (Table 1) with identical settings for both mass spectrometers.

**TABLE 1:** List of tested instrumental settings.

Setting	Default method	Tested values	Comment
Step size	0.02	0.01, 0.04, 0.1	Movement of isolation window centre between fragmentation events in *m/z*
Isolation window width	1	0.4, 0.7, 2	The range of *m/z* values that are isolated for fragmentation
Mass resolving power (R)	120 000	15 000 30 000 60 000	As given by manufacturer
Number of microscans	5	1, 3	Number of microscans averaged per one recorded scan
Collision energy	35–45‐55 stepped	35, 50, 65	Normalised collision energy (NCE)
AGC	5e^4^/default*	2e^4^, 1e^5^/50%, 200%*	Sets the number of ions forwarded to the Orbitrap by controlling the injection time in the C‐trap

* Orbitrap Exploris 120.

### 2.3 Data Processing and Evaluation

All spectra were firstly centroided and transformed into mzML format using msConvert by ProteoWizard [29]. The further data processing was performed in RStudio (version 4.2.3) using packages MSbox, mzR [30], msData [30], ggplot2 [31], stringr [32], fuzzyjoin, tidyr [33], dplyr [34] and OrgMassSpecR [35].

The processing of fragmentation spectra of reference compounds involved several steps. First, the data were centroided by msConvert. SIRIUS 5 [36] was used to annotate the fragmentation spectra with known molecular formulas. The subformulae of fragments were assigned according to the fragmentation tree. These molecular formulas were used to calculate theoretical *m/z* values for further recalibration of the *m/z* values. Peaks with intensities below 0.3% of the highest peak were discarded. The processed reference spectra served as an in‐house library to evaluate the deconvolution of chimeric spectra.

The chimeric fragmentation spectra recorded with 17 prepared instrumental methods were processed as follows. All information of a centroided mzML file containing multiple fragmentation scans was first read into R using the package mzR. To build a template of the chimeric spectrum, the scan with the highest intensity was picked and merged with its 10 neighboring scans (five scans before and five scans after). All scans to be merged were filtered by a ground noise threshold, which was 0.3% of the highest intensity. Peaks in the blank with intensity over 1% of the highest intensity were removed from the template. The blank‐subtracted template was used to build an intensity matrix containing the intensities of each peak from the template for each fragmentation scan. Finally, a correlation matrix was calculated for all pairs of peaks in the intensity matrix, and this matrix was used to deconvolute the chimeric spectra.

The approach to deconvolute chimeric spectra by assigning fragment peaks based on their modulated peak intensity was proposed by Nikolskiy [12] and implemented in a DI‐MS2 workflow by Kaeslin and Zenobi [19], who applied regression‐based techniques. Following this idea, we matched fragments to their precursors (i.e., isobaric compounds) in the chimeric spectra by comparing the correlation score between each fragment and two precursor ions (Figure 1). We used a simple correlation‐based method because our goal was to assess how well precursors correlate with related and unrelated fragments under different instrumental settings. By assigning the fragment to the precursor with the higher correlation score, we reconstructed individual fragmentation spectra from the chimeric spectra. If a precursor ion was missing from the MS2 spectra due to full fragmentation or insufficient resolving power, we used its most intense known fragment as a substitute. However, this simple deconvolution method may not work well if the same fragment is produced by both precursors (Table S5), as it will be only assigned to the precursor with the higher correlation score, decreasing the recall value (see below) of the other precursor. The reconstructed individual fragmentation spectra were then aligned with corresponding reference spectra from our in‐house library, recorded at the same NCE with maximum allowed mass error between theoretical *m/z* and measured *m/z* of 3 mDa. The theoretical *m/z* values from the reference spectra were used to recalibrate *m/z* values in the pseudo‐individual spectra to improve mass accuracy.

The quality of reconstructed fragmentation spectra was evaluated as follows. First, the spectral similarity between recalibrated pseudo‐individual fragmentation spectra *u* and reference fragmentation spectra *v* was calculated using a mass error of 0.2 mDa threshold to align *m/z* ratios in both spectra using the OrgMassSpecR package:

(1) $$\text{Similarity score}=\frac{\left\langle u,v\right\rangle }{\sqrt{\sum \left\langle u,u\right\rangle }\cdot \sqrt{\sum \left\langle v,v\right\rangle }},\text{where}\;\left\langle x,x\right\rangle \;\text{is}\;a\;\mathrm{dot}\;\text{product}$$

Secondly, the precision of reconstructed fragmentation spectra was calculated to evaluate how correctly peaks were assigned to reconstructed spectra:

(2) $$\text{Precision}=\frac{\sum \text{Intensity of correctly assigned peaks in reconst}.\;\mathrm{MS}2}{\sum \text{Intensity of}\;\mathrm{all}\;\text{peaks in reconstr}.\;\mathrm{MS}2}$$

It is important to note that the deconvolution of chimeric spectra is successful when the precision of a reconstructed spectrum is high for both isobars, as the precision is also high when only one peak is correctly assigned and the rest of the peaks are missing from a reconstructed spectrum. The precision decreases only when peaks are incorrectly assigned to a reconstructed spectrum, that is when peaks assigned to a reconstructed spectrum are not found in the corresponding reference spectrum.

Finally, recall of reconstructed fragmentation spectra was calculated to evaluate what fraction of peaks was correctly reconstructed as compared with reference spectra:

(3) $$\text{Recall}=\frac{\sum \text{Intensity of correct}.\;\text{assigned in reconst}.\mathrm{MS}2\;\mathrm{as}\;\mathrm{per}\;\mathrm{ref}.\mathrm{MS}2}{\sum \text{Intensity of}\;\mathrm{all}\;\text{in}\;\mathrm{ref}.\;\mathrm{MS}2}$$

As with precision, the deconvolution of chimeric spectra is successful when recall is high for both isobars. Recall is high when all peaks present in the reference fragmentation spectrum are assigned to the reconstructed spectrum. However, the presence of multiple incorrectly assigned peaks in a reconstructed spectrum does not decrease its recall value.

## 3 Results and Discussion

### 3.1 Differences in Signal Intensity Modulation by Different Mass Spectrometers

**FIGURE 3:**
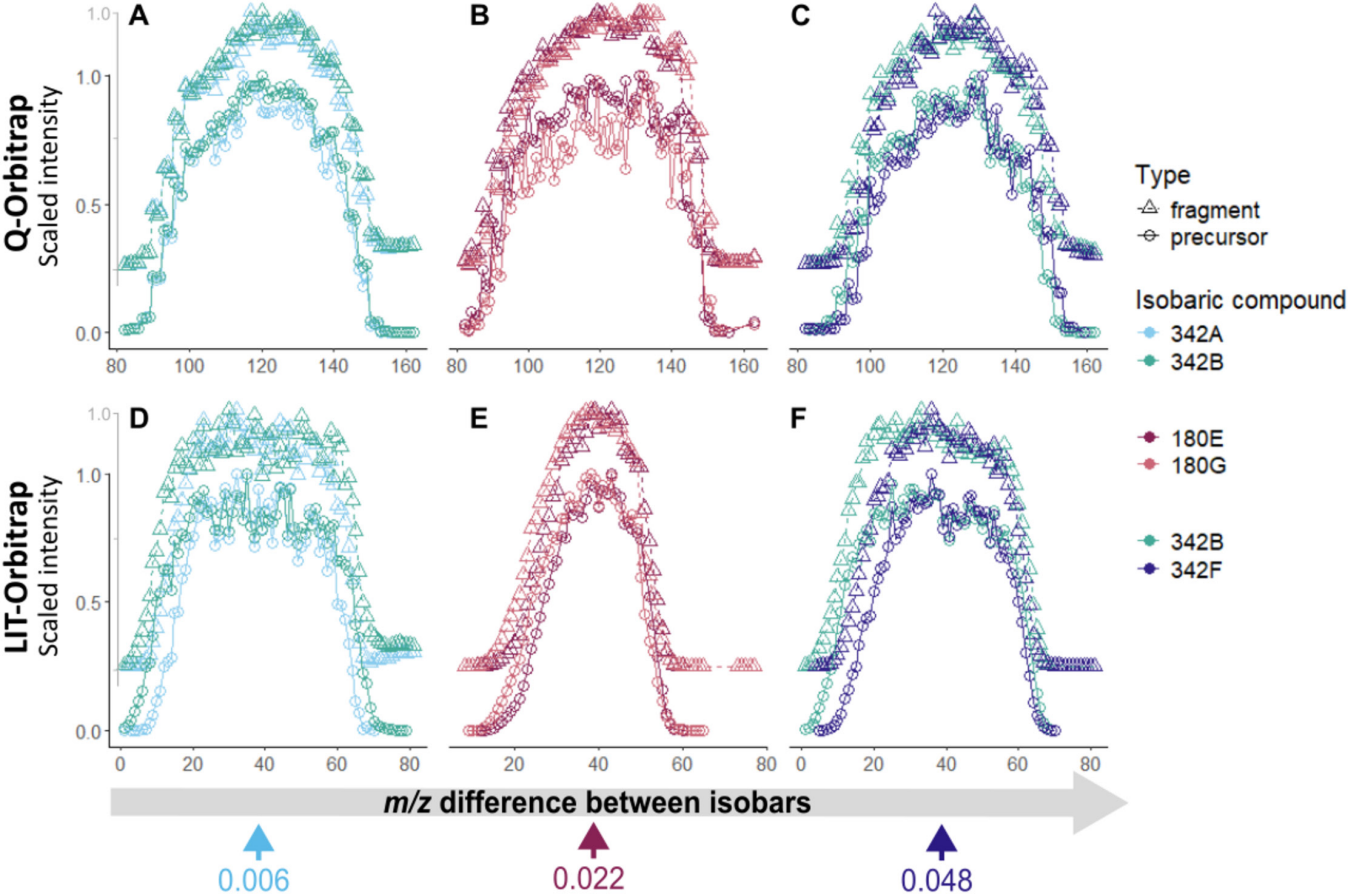
Profiles of the modulated precursors' and their main fragments' intensities observed for different isobaric mixtures under default instrumental settings recorded using Q‐Orbitrap (upper row, A, B, C) and LIT‐Orbitrap (lower row, D, E, F): (A, D) mixture of isobars 342A + 342B; (B, E) mixture of isobars 180E + 180G; (C, F) mixture of isobars 342B + 342F. The intensity of fragments is offset by 0.25 for better visibility. Note the unexpected profile patterns for LIT‐Orbitrap.

Based on the principle of stepwise movement of the isolation window, we expect the modulated intensities of ions with different *m/z* to be shifted in relation to each other: When the isolation window shifts from low *m/z* to high, signals from precursors with smaller *m/z* values appear earlier and disappear sooner in the fragmentation spectra than those from precursors with larger *m/z* values. (Figure 1). This behaviour has been previously observed for a Q‐Orbitrap instrument [19] and was confirmed in our study (Figure 3C; Figure S1). When the step size between scans is constant, the distance between the modulated intensity profiles of different isobars depends on their *m/z* difference and, consequently, isobars with closer *m/z* values will show more similar modulated intensity profiles (Figure S2). For example, the modulated intensities overlap for isobars with closest *m/z* values, that is, 342A + 342B (Figure 3A), 318B + 318C, and 302B + 302D (Figure S1). Conversely, isobars with larger *m/z* differences, like 342B + 342F (Figure 3C) and 318A + 318F (Figure S1), exhibited more distinct shifts in intensity profiles.

Unexpectedly, the LIT‐Orbitrap instrument showed a different pattern. Here, modulated intensities varied between isobars, regardless of their *m/z* differences. Both isobars with the closest and farthest *m/z* ratios exhibited distinctly different modulated intensities (Figure 3D,F). Unlike in the Q‐Orbitrap instrument (Figure 3C), where signals of the lighter isobar appeared and disappeared first in MS2 spectra, the LIT‐Orbitrap data often showed a ‘widening’ pattern, where one isobar's modulated intensity appeared first in MS2 spectra but disappeared last throughout a measurement cycle. This pattern was consistent across multiple isobaric mixtures (Figure S1).

Generally, larger *m/z* differences between isobars resulted in greater shifts in modulated intensities on the Q‐Orbitrap instrument, while the LIT‐Orbitrap modulated intensities were independent of *m/z* differences (Figure S2). This suggests that the LIT‐Orbitrap may effectively separate isobars with very close *m/z* values, though the exact reasons for these modulation differences remain unclear, complicating predictions for chimera deconvolution performance. The tendency of a compound to fragment may be one of the properties associated with the shape of its modulated intensity profile on the LIT‐Orbitrap, which determines shifts in modulated intensity between compounds. To assess this, we estimated an NCE50, defined as the NCE at which the precursor ion accounts for 50% of the total MS2 signal intensity (Table S6). A higher NCE50 indicates a compound that is more resistant to fragmentation. Compounds with higher NCE50 tended to exhibit broader modulated intensity profiles in an isobaric mixture. We hypothesise that molecules with lower NCE50 might fragment more readily during resonance ejection of unwanted ions out of LIT, resulting in greater signal loss and narrower intensity profiles. However, the link between NCE50 and intensity modulation is not perfect. Moreover, the shape of the modulated intensity profile did not appear to depend on the relative intensity of the precursor ion (Figure S8) or its collision cross‐section (Table S6).

Since the deconvolution of chimeric spectra relies on differences in modulated intensities, the distinction between mass spectrometric platforms could impact the deconvolution outcome under varying instrumental conditions and will be discussed in the following sections.

### 3.2 Quality of Deconvoluted MS2 Spectra

**FIGURE 4:**
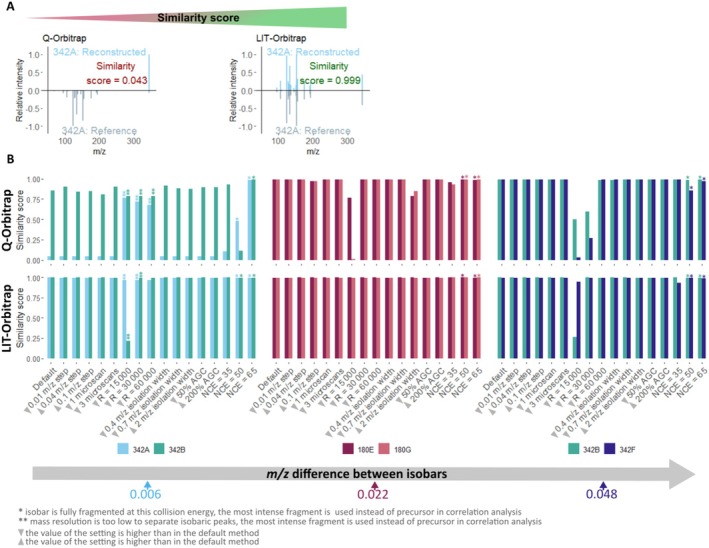
(A) Example of mirror plots comparing an isobar's reconstructed individual MS2 spectrum and the respective reference spectrum (here, compound 342A) with corresponding similarity scores (0—dissimilar, 1—similar). Data were recorded with default settings. (B) Similarity scores between reference spectra and reconstructed individual fragmentation spectra of isobaric compounds obtained under different instrumental settings on Q‐Orbitrap (upper row) and LIT‐Orbitrap (lower row). Different colours denote different isobaric compounds.

**FIGURE 5:**
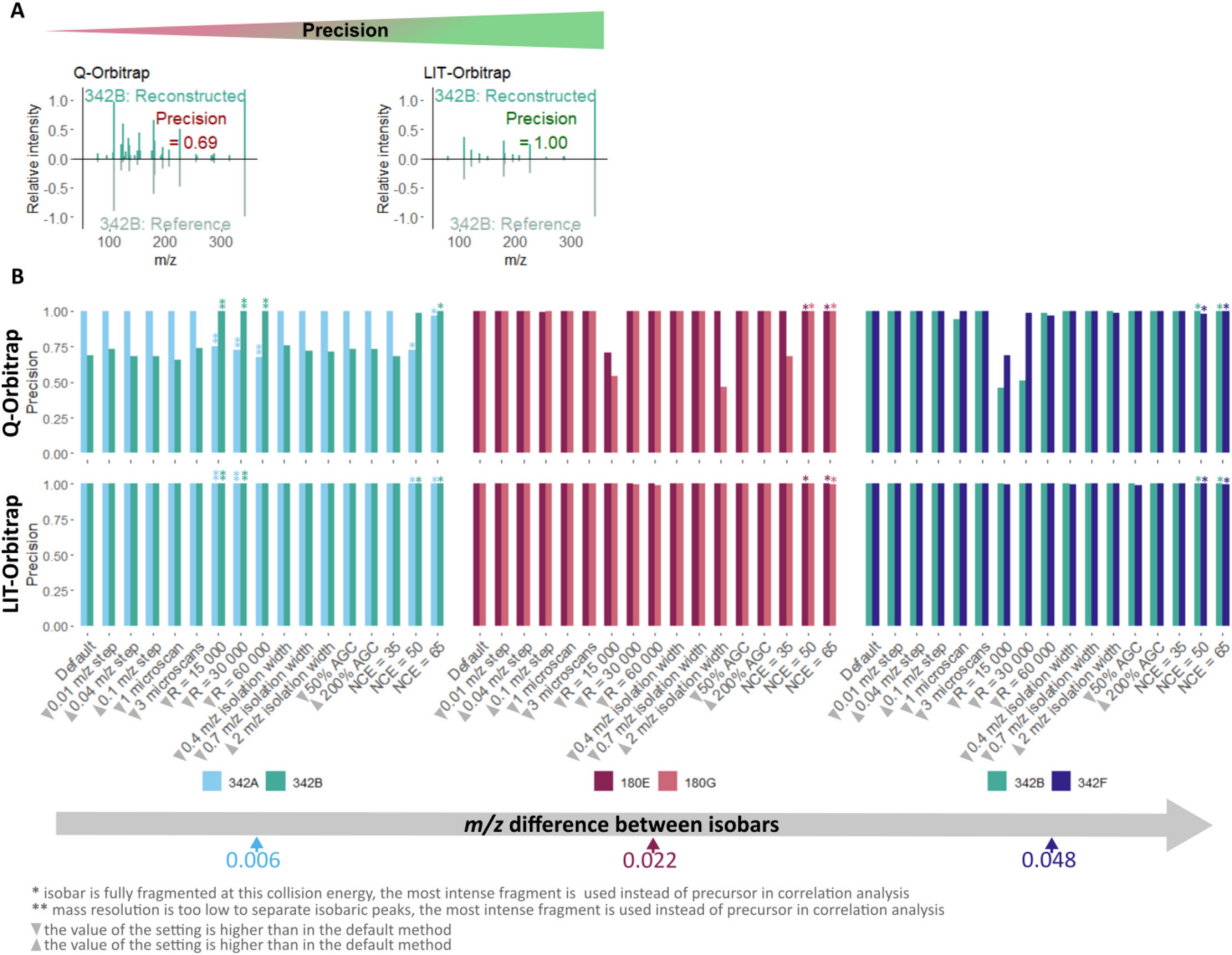
(A) Example of mirror plots comparing an isobar's reconstructed individual MS2 spectrum and the respective reference spectrum (here, compound 342B) with corresponding precision. (0—all peaks assigned to a reconstructed spectrum are missing from the reference spectrum, i.e., only incorrect assignments, 1—all assigned peaks are present in reference spectra). Data were recorded with default settings. (B) Precision of reconstructed individual fragmentation spectra obtained under different instrumental settings on Q‐Orbitrap (upper row) and LIT‐Orbitrap (lower row). Different colours denote different isobaric compounds.

Despite the differences in intensity modulation and the unexpected behaviour of the LIT‐Orbitrap, the method successfully deconvoluted chimeric MS2 spectra of isobaric mixtures on both the LIT‐Orbitrap and Q‐Orbitrap instruments. For the LIT‐Orbitrap, changes in instrumental settings had little impact on the deconvolution quality with average similarity scores between reference MS2 spectra and reconstructed individual MS2 spectra of 0.98, with only a few scores below 0.97. High similarity scores were consistently achieved across all isobaric mixtures, including the one with the closest *m/z* values, that is 342A + 342B (Δ*m/z* = 0.006), and under various instrumental settings (Figure 4). The only exception was the lowest mass resolving power of 15 000, which led to a low similarity score for the MS2 spectra of compound 342B reconstructed from isobaric mixtures 342A + 342B and 342B + 342F. However, the decrease in similarity scores was due to the decreased mass accuracy rather than incorrect peak assignment, as all reconstructed spectra were characterised by high precision (Figure 5B and Table S3). Otherwise, contrary to our expectations, we observed a high quality of deconvoluted chimeric spectra independent of the used instrumental settings and mass differences between isobars' *m/z* values on the LIT‐Orbitrap.

In contrast, the performance of the Q‐Orbitrap varied more strongly between different isobaric pairs and instrumental settings. As anticipated, high‐quality deconvolution of chimeric spectra was achieved for isobaric pairs with higher *m/z* differences, such as 180E + 180G and 342B + 342F, reaching an average similarity score of 0.96 (Figure 4). However, for the isobaric pair with the smallest *m/z* difference of 0.006, 342A + 342F, the average similarity score dropped to 0.56 (Figure 4). Mass resolving power was one of the most influential parameters for both instruments; however, unlike the LIT‐Orbitrap, the choice of low resolving power on the Q‐Orbitrap did not only decrease the similarity scores but also resulted in the incorrect assignment of peaks to reconstructed individual fragmentation spectra leading to low precision (Figure 5 and Table S3).

The lowest quality of reconstructed individual fragmentation spectra was observed for the isobaric pair with the smallest mass difference 342A + 342B (Δ*m/z* = 0.006) on the Q‐Orbitrap. Under all tested settings, the reconstructed fragmentation spectra had erroneously assigned peaks leading to a decrease in precision (Figure 5B and Table S3). When both precursors were present in MS2 spectra, the similarity score for compound 342A was only 0.04 (Figure 4), with all fragments incorrectly assigned to compound 342B (Supplementary Figure S3). This likely resulted from the lower intensity of the precursor peak 342A compared with precursor peak 342B with an intensity ratio of about 1:20 at stepped collision energy 35‐45‐55 NCE. However, when one or both precursor peaks were absent from MS2 spectra due to low mass resolution (*R* = 15 000, 30 000 and 60 000), or high collision energy (NCE 50 and 65), and the corresponding most intense fragment was used as precursor for correlation, the similarity score increased to an average of 0.82 and the number of incorrectly assigned peaks decreased (Table S3). This suggests that the reason for the low quality of spectra deconvolution on the Q‐Orbitrap is twofold. Firstly, it stems from the close masses of precursors, because some peaks were incorrectly assigned in all cases, even when precursors were substituted by fragments for deconvolution. Secondly, the intensities of precursor peaks play an important role, too: When the less intense precursor 342A or both precursors 342A and 342B were substituted by their most intense fragments, the deconvolution quality improved. Low intensity leads to higher signal variation throughout the measurement cycle, and very low intensity peaks can be missing or removed as noise in some of the MS2 scans. Since the reconstruction of individual MS2 spectra depends on the correlation between fragments and precursor signals across the measurement cycle, high signal variation may decrease the correlation values. A high intensity fragment has a more stable signal over the measurement cycle, so if it is used as a substitution for a precursor, the correlation values can improve.

Contrary to our assumption, the step size between scans had no noticeable impact on method performance on both instruments. No peaks were assigned incorrectly with large step sizes on the LIT‐Orbitrap, and the step size effect observed on the Q‐Orbitrap was not strong: When the largest step size of 0.1 *m/z* was used, the precision of reconstructed fragmentation spectra remained high, as only one fragment was incorrectly assigned to compound 180E and to compound 342B from the mixture 342B + 342F (Figure 5B; Table S3). Thus, a step size much bigger than the mass difference between isobars can be used to successfully reconstruct individual MS2 spectra from chimeric spectra on both instruments. However, the chimeric spectra of isobars with very close masses (342A + 342B; Figures 4 and 5) were difficult to deconvolute on Q‐Orbitrap even when using the smallest step size of 0.01 *m/z*.

Collision energy strongly influences the quality of fragmentation spectra, which, in turn, can impact method performance. The use of too high collision energy leads to the disappearance of precursor peaks from the fragmentation spectra. If a fragment of one of the precursors is known, it can be used instead of the missing precursor to group fragments into a reconstructed individual fragmentation spectrum, as we did in this study; however, such information is usually not available for an unknown mixture. Therefore, the choice of collision energy is very important for the deconvolution of chimeric spectra and should be adapted to the sample. Generally, stepped collision energy is recommended for complex samples: It produces a wide range of fragments and allows preservation of the precursors' signal in fragmentation spectra, simplifying the deconvolution of chimeric MS2, thus serving as a good starting point for analysis [24, 37, 38].

As for the isolation window width, using narrower windows (e.g., 0.4 or 0.7 *m/z*) was beneficial, as it reduced the number of fragmentation steps needed to modulate precursor signals compared with the default 1 *m/z* window width, without compromising deconvolution quality. The use of a wider isolation window is not recommended, as it led to incorrect assignment of multiple peaks on the Q‐Orbitrap (e.g., isobaric mixtures 180E + 180G and 342B + 342F; Figure 4B; Table S3) and reduced precursor‐fragment correlation on both instruments (Figure S5). More ions, including isotopic ions of target precursors [M + H + 1]^+^ and [M + H + 2]^+^, are co‐isolated by a wide isolation window. Isotopic ions can produce the same fragments as target precursors, and these fragments would appear in the fragmentation spectra at the end of the measurements cycle, when the centre of the isolation window is far from the target precursor's *m/z* and close to the *m/z* of its isotopologue (Figure S6). As a result, the correlation of signal intensities between a target precursor and its fragments would decrease, resulting in lower deconvolution quality. Moreover, a wider window requires more fragmentation steps to modulate precursor intensities, increasing the analysis time.

The number of microscans per scan and the automatic gain control (AGC) target had a minor impact on the quality of chimeric spectra deconvolution. Reducing the number of microscans generally did not affect the similarity scores of reconstructed MS2 spectra but caused a slight decrease in the correlation between precursors and fragments (Figure S5). In one case, the latter resulted in an incorrect assignment of several fragments using one microscan per scan on the Q‐Orbitrap (compound 342B from the 342B + 342F mixture). Similarly, reducing the AGC target slightly reduced the correlation between precursors and fragments (Figure S5), which led to one low‐intensity fragment being erroneously assigned to the wrong isobar on the LIT‐Orbitrap instrument (compound 342F from the mixture 342B + 342F at 50% AGC; Figure 5B and Table S3). Although reducing the AGC target value has been reported to improve mass accuracy by reducing space‐charge effects in the Orbitrap cell [26], we observed similar mass errors within the range of AGC target values used in this study (Figure S4). These observations suggest that both parameters can be adjusted without substantially affecting the deconvolution of chimeric spectra. The number of microscans can be reduced to one or three for shorter analysis time. With the instrument's default AGC setting being a good starting point, the AGC value can be adjusted to improve sensitivity or mass accuracy [24, 25, 39].

In summary, we found that mass resolving power was the most influential instrumental parameter for both platforms. Isobaric peaks are not separated under inadequate mass resolution, which leads to the absence of true precursor peaks from mass spectra, as seen with the isobaric mixture 342A + 342B at lower mass resolving power (Figure S7). The same applies for fragments: If co‐fragmented compounds produce similar fragments, for example, through the same neutral loss, these peaks might not be separated as well, leading to decreased mass accuracy. In this study, when isobaric peaks were not separated, we could use the most intense fragments as a proxy for a precursor to reconstruct the individual MS2 spectra, which would be impossible for an unknown mixture. Therefore, the choice of medium to high mass resolving power is advised for very complex mixtures such as, for example, dissolved organic matter [40] and petroleum [41], or for isobars with higher *m/z* values that require higher mass resolution and likely have complex fragmentation spectra. Moreover, the choice of appropriate collision energy that produces enough informative fragments and retains precursors in MS2 spectra is important for the method performance, especially for mixtures with unknown properties or when correlation between precursors and fragments is used to deconvolute chimeric spectra, as the intensity of precursors in MS2 should be high enough to produce a stable signal across fragmentation scans. The influence of step size on deconvolution quality was minimal, with lower step sizes providing slightly better deconvolution quality on the Q‐Orbitrap. Wide isolation window width should be avoided as it decreased the deconvolution quality and increased analysis time. The number of microscans and AGC target value had a weaker impact, but can be optimised to improve MS2 spectral quality if needed.

### 3.3 Analysis Time

**FIGURE 6:**
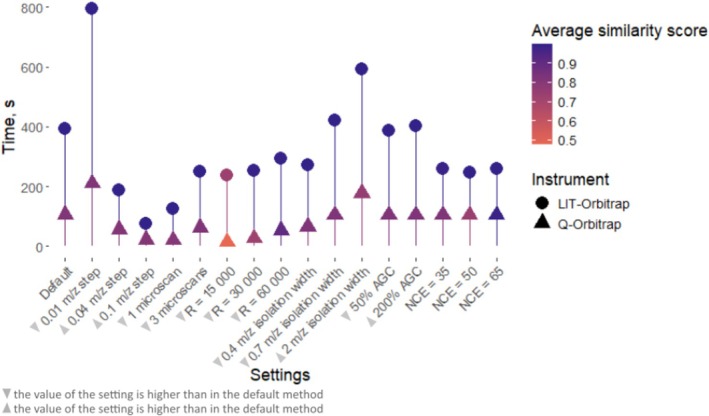
Length of one measurement cycle under different instrumental settings. The colour corresponds to the similarity score between reconstructed individual and corresponding reference spectra averaged over all six isobars from three isobaric mixtures measured with given settings.

The Q‐Orbitrap platform allowed for ca. four times shorter analysis time compared with the LIT‐Orbitrap, lowering the average run time from 320 to 85 s (Figure 6 and Table S4). Methods with the highest number of fragmentation events, such as those using the smallest step size (*m/z* 0.01) and the widest isolation window (*m/z* = 2), were the most time‐consuming. Despite providing more scans for correlating precursors and fragments, these methods did not improve the deconvolution quality (Figures 4 and 5). Conversely, methods with fewer fragmentation events, achieved by using larger step sizes or narrower isolation windows, did not reduce the deconvolution quality despite reducing the analysis time significantly. For example, using the largest step size (0.1 *m/z*) on the LIT‐Orbitrap reduced analysis time fivefold, from 390 s using the default method to 76 s without hindering the reconstruction of individual MS2 spectra. On the Q‐Orbitrap, an erroneous assignment of low intensity peaks to reconstructed MS2 spectra was observed at the largest step size, while the step size of 0.04 *m/z* did not compromise method performance at all while halving the run time. Similarly, reducing the isolation window width to *m/z* 0.4 did not undermine the method performance yet decreased analysis time by approximately 30% on both instruments (from 390 to 271 s on LIT‐Orbitrap and from 105 to 66 s on Q‐Orbitrap). However, the use of a smaller isolation window decreases sensitivity [12] and should therefore be used with caution for fragmentation of low intensity analytes.

Lowering of mass resolution expectedly decreased the analysis time. Interestingly, this effect was more pronounced for the Q‐Orbitrap: Using a resolving power of 15 000 made the measurements about nine times faster compared with a resolving power of 120 000 in the default method, whereas the LIT‐Orbitrap was only about 1.7 times faster under the same settings. Despite this, we generally advise against using low mass resolution for complex samples, as it led to wrong peak assignments on the Q‐Orbitrap, decreased mass accuracy on the LIT‐Orbitrap and resulted in complete overlap of isobaric peaks for mixture 342A + 342B (Figure S7).

Reducing the number of microscans per scan generally did not affect the similarity scores between pseudo‐individual and reference spectra but strongly reduced analysis time. On the LIT‐Orbitrap, analysis time decreased threefold from an average of 390 s with default settings to 125 s with one microscan; on the Q‐Orbitrap—five times, from an average of 105 to 21 s.

The use of individual collision energies instead of the stepped one did not change the run time on the Q‐Orbitrap but did so on the LIT‐Orbitrap. On the LIT‐Orbitrap, stepped NCE needed about 1.5 times longer compared with the use of a single NCE (390 and 255 s respectively). Nonetheless, we recommend using stepped collision energy on an LIT‐Orbitrap instrument as well, unless the fragmentation behaviour of the sample is known.

Since the AGC target determines the number of ions that are accumulated before being sent to the Orbitrap analyser, a lower AGC target can potentially speed up the analysis. Indeed, using a 50% AGC value slightly shortened the measurement time, but only for the LIT‐Orbitrap (Table S4), without affecting the quality of the reconstructed fragmentation spectra. Therefore, while decreasing AGC may not significantly improve the analysis time, it can decrease sensitivity and should be optimised depending on the sample.

### 3.4 Final Recommendations

To optimise analysis time without compromising the quality of chimeric spectra deconvolution across different MS platforms, we recommend using the DI‐MS2 method with medium or high mass resolving power, one to three microscans per scan, default AGC target values and stepped collision energy. Application of a narrower isolation window (e.g., 0.4 *m/z*) can further decrease the analysis time by decreasing the *m/z* range that needs to be scanned.

Despite unexpected patterns of intensity modulation on the LIT‐Orbitrap instrument, effective deconvolution of chimeric MS2, even for isobars with the closest masses (342A + 342B), was achieved across all tested settings, which provides the user with more flexibility in the implementation of the method. The analysis time can be significantly improved by using wider step sizes between consecutive fragmentation scans. The ability to separate isobars even with a small *m/z* difference of 0.006 using a 0.1 *m/z* step size likely results from unexpected differences in their modulated intensities, which are not observed with the Q‐Orbitrap instruments (Figure 3). Therefore, the LIT‐Orbitrap instrument can be effectively used under diverse settings even for complex mixtures, as long as the modulated intensities of precursors are distinct. The reasons for the unexpected modulation behaviour (shifts in precursor ion abundances due to moving isolation window) of the LIT‐Orbitrap remain to be investigated in detail.

When applying the DI‐MS2 method on a Q‐Orbitrap instrument, larger step sizes do not necessarily compromise the deconvolution quality too. Only one peak was incorrectly assigned to reconstructed individual spectra for mixtures 180E + 180G and 342B + 342F measured with the largest step size *m/z* 0.1. Thus, we recommend starting with medium step sizes (between 0.04 and 0.1 *m/z*) to save analysis time without compromising deconvolution quality. However, it should be noted that the isobars with closest *m/z* 342A + 342B (Δ*m/*z = 0.006) were not well separated on the Q‐Orbitrap even when the smallest step size *m/z* 0.01 was used.

Additionally, the intensity of precursor signals in MS2 plays an important role as well, especially when correlation between precursor and fragment signals is used to reconstruct spectra, as performed in this study. The deconvolution quality could be improved by substituting low‐intensity precursor signals with their higher‐intensity fragments to calculate the correlation score, as observed for the 342A + 342B mix on the Q‐Orbitrap at reduced mass resolution or increased collision energy. This approach however is likely not applicable to unknown mixtures as the association of ions with isobars is unknown. Therefore, if signals of precursors are used for spectra deconvolution, it is more likely to produce high‐quality reconstructed MS2 spectra when the intensities of precursor peaks in MS2 are high enough to produce a stable signal in MS2 scans.

## 4 Conclusions

Neighbouring precursor ions with small mass differences (isobars) can obscure MS2 fragmentation spectra, leading to so‐called ‘chimeric’ MS2 spectra. A DI‐MS2 method that modulates intensities by stepwise movement of the quadrupole isolation window had been successfully used to deconvolute chimeric fragmentation spectra in the individual MS2 spectra. This study evaluated its implementation across two different MS platforms, a LIT‐Orbitrap and Q‐Orbitrap, to assess the method's versatility and the influence of instrumental settings on the analysis time and deconvolution quality.

Our findings demonstrate that the discussed DI‐MS2 method can be successfully implemented on both quadrupole‐based and ion trap‐based instruments. Notably, the character of intensity modulation upon moving the isolation window was different between LIT‐ and Q‐Orbitraps: The LIT‐Orbitrap produced distinct intensity modulation patterns, even for isobaric compounds with very close masses (Δ*m/z* = 0.006), enabling high‐quality deconvolution of their chimeric fragmentation spectra. Despite these differences between tested instruments, individual MS2 spectra were effectively reconstructed from chimeric MS2 spectra on both platforms.

The LIT‐Orbitrap provided robust and flexible performance under a wide range of settings regardless of *m/z* differences between analysed isobars. In contrast, the Q‐Orbitrap, offered significantly shorter analysis time but showed more variability in deconvolution quality, particularly for isobars with the closest *m/z* values (Δ*m/z* = 0.006).

For both instruments, mass resolution was an important parameter, so the use of medium or high mass resolving power is recommended. Surprisingly, step size had little impact on the deconvolution quality: even the largest step size of 0.1 *m/z* allowed for successful spectra deconvolution on both instruments. Therefore, to improve analysis time without compromising deconvolution quality, a rather large step size of 0.1 *m/z* can be used. The choice of a smaller step size, for example, 0.04 *m/*z, is a safer option for Q‐Orbitrap instruments to avoid erroneously assigned peaks. Additionally, the choice of narrow fragmentation windows (0.4 or 0.7 *m/z*), stepped NCE, default AGC target value and averaging of one to three microscans per scan can shorten run time without affecting method performance. Finally, better quality of chimeric spectra deconvolution is achieved when the intensities of precursor peaks in MS2 are high enough to produce stable signal in MS2 scans, at least when correlation between precursors and fragments is used in deconvolution.

With optimised settings, the DI‐MS2 method can be used to scan several tens of nominal masses within 20–30 min, potentially yielding fragmentation spectra of hundreds of compounds. This capacity would provide valuable insight into the structural composition of complex biological samples, complementing established chromatography–tandem mass spectrometry methods.

## Author Contributions

Arina Ivanova: writing – original draft, methodology, investigation, data curation. Wei Tang: methodology, writing – review and editing. Carsten Simon – writing – review and editing. Kai Dührkop – methodology, supervision. Sebastian Böcker: methodology, supervision, funding acquisition, writing – review and editing. Gerd Gleixner: conceptualisation, supervision, funding acquisition, writing – review and editing.

## Conflicts of Interest

The authors declare no conflicts of interest.

## Supporting information


**Table S1:** Properties of used compounds.
**Table S2:** The detailed ESI source settings.
**Table S3:** Number of peaks in each reconstructed pseudo‐individual MS2 spectrum compared with corresponding reference spectrum: in blue—correctly assigned peaks; in grey—missing peaks; in red—incorrectly assigned peaks that belong to the other isobar in isobaric mixture.
**Table S4:** Length of one measurement cycle under different instrumental settings.
**Table S5:** Shared fragmentation peaks of isobaric mixtures.
**Table S6:** Estimated NCE50 (normalised collision energy at which the precursor ion accounts for 50% of the total MS2 signal intensity), predicted collision cross section* and width of modulated intensity profiles for three isobaric mixtures.
**Figure S1:** Shapes of the modulated precursors' intensities observed for different isobaric mixtures measured at 35 NCE with otherwise default settings, Δ*m/z* corresponds to the difference between *m/*z values of isobars' [M + H] + ions. The top row recorded with LIT‐Orbitrap and bottom row recorded with Q‐Orbitrap. While there is no clear connection between Δ*m/z* values and the difference between isobars' modulated intensities for LIT‐Orbitrap, the bigger Δ*m/z* generally lead to larger shift between modulated intensities for Q‐Orbitrap.
**Figure S2:** Distance between modulated intensity profiles for isobaric pairs from Figure 3 and Figure S1 changing with *m/z* differences between isobars' [M + H] + ions (right). The distance between modulated intensities is calculated as a number of scans between the MS2 events, in which one of isobars reaches relative intensity of 0.5 (e.g., if precursor A reaches 0.5 relative intensity at scans 30 and 60 and precursor B—at scans 35 and 65, then the distance between modulated intensity profiles of isobars A and B is |35–30| + |65–60| = 10 scans; left). The data were measured at 35 NCE with otherwise default settings. While there is no clear connection between *m/z* differences and the distance between isobars' modulated intensities for LIT‐Orbitrap, there is a weak trend for Q‐Orbitrap: Bigger *m/z* differences lead to larger shift between modulated intensities.
**Figure S3: (**A) Example of mirror plots comparing an isobar's reconstructed individual MS2 spectrum and the respective reference spectrum (here, compound 342A) with corresponding recall. (0—no peaks present in the reference spectrum were assigned to a reconstructed spectrum; i.e., no peaks were correctly assigned; 1—all peaks present in reference spectrum were assigned to a reconstructed spectrum). Data were recorded with default settings. (B) Recall of reconstructed individual fragmentation spectra obtained under different instrumental settings on Q‐Orbitrap (upper row) and LIT‐Orbitrap (lower row). Different colours denote different isobaric compounds. * isobar is fully fragmented at this collision energy; the most intense fragment is used instead of precursor in correlation analysis; ** mass resolution is too low to separate isobaric peaks; the most intense fragment is used instead of precursor in correlation analysis. Δ represents that the value of the setting is higher than in the default method; ∇ indicates that the value is lower.
**Figure S4:** Average mass error before recalibration at different AGC values for three tested isobaric mixtures and two mass spectrometers. Only measurements performed at the same day are considered to exclude day‐to‐day variation in mass accuracy. Error bars are given at one standard deviation level.
**Figure S5:** Average correlation value between fragments and precursors obtained under different instrumental settings on LIT‐Orbitrap and Q‐Orbitrap. The correlation value for each setting is averaged over all six isobars from three isobaric mixtures.
**Figure S6:** Comparison of intensity modulation profiles of precursors and their most intense fragments when using different isolation window width.
**Figure S7:** The sections of MS1 spectra of isobaric mixture 342A + 342B recorded at mass resolution 480 000 (left) and 30 000 (right) on LIT‐Orbitrap. The individual isobaric peaks cannot be separated at mass resolution below 60 000 for LIT‐Orbitrap and 120 000 for Q‐Orbitrap.
**Figure S8:** Modulated precursors' intensity profiles observed for the same isobaric mixture at different concentrations. Compound 318D has ‘wider’ modulated intensity profile also when its intensity is lower than intensity of compound 318A (upper row).

## Data Availability

The method files and centroided spectra can be found in the Edmund repository under https://doi.org/10.17617/3.XIWDCQ. The scripts used for data processing and visualisation can be found in GitHub under https://github.com/arina‐iva/DI‐MS2_scripts.

## References

[1] E. E. Kempa , K. A. Hollywood , C. A. Smith , and P. E. Barran , “High Throughput Screening of Complex Biological Samples With Mass Spectrometry—From Bulk Measurements to Single Cell Analysis,” Analyst 144, no. 3 (2019): 872–891, 10.1039/c8an01448e. 30601490

[2] L. Lin , Q. A. Yu , X. M. Yan , et al., “Direct Infusion Mass Spectrometry or Liquid Chromatography Mass Spectrometry for Human Metabonomics? A Serum Metabonomic Study of Kidney Cancer,” Analyst 135, no. 11 (2010): 2970–2978, 10.1039/c0an00265h. 20856980

[3] S. Anand , S. Young , M. S. Esplin , et al., “Detection and Confirmation of Serum Lipid Biomarkers for Preeclampsia Using Direct Infusion Mass Spectrometry,” Journal of Lipid Research 57, no. 4 (2016): 687–696, 10.1194/jlr.P064451. 26891737 PMC4808777

[4] J. Ecker , E. Benedetti , A. Kindt, SD , et al., “The Colorectal Cancer Lipidome: Identification of a Robust Tumor‐Specific Lipid Species Signature,” Gastroenterology 161, no. 3 (2021): 910–923.e919, 10.1053/j.gastro.2021.05.009. 34000281

[5] I. Perkons , J. Rusko , D. Zacs , and V. Bartkevics , “Rapid Determination of Pharmaceuticals Inwastewater by Direct Infusion HRMS Using Target and Suspect Screening Analysis,” Science of The Total Environment 755 (2021): 142688, 10.1016/j.scitotenv.2020.142688. 33059144

[6] H. A. Haijes , M. Willemsen , M. Van der Ham , et al., “Direct Infusion Based Metabolomics Identifies Metabolic Disease in Patients' Dried Blood Spots and Plasma,” Metabolites 9, no. 1 (2019): 12, 10.3390/metabo9010012. 30641898 PMC6359237

[7] K. Zhang , W. Liu , Q. Song , et al., “Integrated Strategy Drives Direct Infusion–Tandem Mass Spectrometry as an Eligible Tool for Shotgun Pseudo‐Targeted Metabolomics of Medicinal Plants,” Analytical Chemistry 93, no. 4 (2021): 2541–2550, 10.1021/acs.analchem.0c04602. 33439008

[8] S. Sidoli , Y. Kori , M. Lopes , et al., “One Minute Analysis of 200 Histone Posttranslational Modifications by Direct Injection Mass Spectrometry,” Genome Research 29, no. 6 (2019): 978–987, 10.1101/gr.247353.118. 31123082 PMC6581051

[9] E. A. Trujillo , A. S. Hebert , J. C. R. Vazquez , et al., “Rapid Targeted Quantitation of Protein Overexpression With Direct Infusion Shotgun Proteome Analysis (DISPA‐PRM),” Analytical Chemistry 94, no. 4 (2022): 1965–1973, 10.1021/acs.analchem.1c03243. 35044165 PMC9007395

[10] N. O. Potocnik , G. L. Fisher , A. Prop , and R. M. A. Heeren , “Sequencing and Identification of Endogenous Neuropeptides With Matrix‐Enhanced Secondary Ion Mass Spectrometry Tandem Mass Spectrometry,” Analytical Chemistry 89, no. 16 (2017): 8223–8227, 10.1021/acs.analchem.7b02573. 28753276 PMC5566790

[11] I. Lanekoff , K. Burnum‐Johnson , M. Thomas , et al., “High‐Speed Tandem Mass Spectrometric in Situ Imaging by Nanospray Desorption Electrospray Ionization Mass Spectrometry,” Analytical Chemistry 85, no. 20 (2013): 9596–9603, 10.1021/ac401760s. 24040919 PMC3867692

[12] I. Nikolskiy , N. G. Mahieu , Y. J. Chen , R. Tautenhahn , and G. J. Patti , “An Untargeted Metabolomic Workflow to Improve Structural Characterization of Metabolites,” Analytical Chemistry 85, no. 16 (2013): 7713–7719, 10.1021/ac400751j. 23829391 PMC3983953

[13] L. Liu , C. X. Song , S. B. Tian , et al., “Structural Characterization of Sulfur‐Containing Aromatic Compounds in Heavy Oils by FT‐ICR Mass Spectrometry With a Narrow Isolation Window,” Fuel 240 (2019): 40–48, 10.1016/j.fuel.2018.11.130.

[14] R. A. Scheltema , J. P. Hauschild , O. Lange , et al., “The Q Exactive HF, a Benchtop Mass Spectrometer With a Pre‐Filter, High‐Performance Quadrupole and an Ultra‐High‐Field Orbitrap Analyzer,” Molecular & Cellular Proteomics 13, no. 12 (2014): 3698–3708, 10.1074/mcp.M114.043489. 25360005 PMC4256516

[15] T. I. Roumeliotis , H. Weisser , and J. S. Choudhary , “Evaluation of a Dual Isolation Width Acquisition Method for Isobaric Labeling Ratio Decompression,” Journal of Proteome Research 18, no. 3 (2019): 1433–1440, 10.1021/acs.jproteome.8b00870. 30576155 PMC6399672

[16] M. Lísa , E. Cífková , M. Khalikova , M. Ovčačíková , and M. Holčapek , “Lipidomic Analysis of Biological Samples: Comparison of Liquid Chromatography, Supercritical Fluid Chromatography and Direct Infusion Mass Spectrometry Methods,” Journal of Chromatography a 1525 (2017): 96–108, 10.1016/j.chroma.2017.10.022. 29037587

[17] S. Houel , R. Abernathy , K. Renganathan , K. Meyer‐Arendt , N. G. Ahn , and W. M. Old , “Quantifying the Impact of Chimera MS/MS Spectra on Peptide Identification in Large‐Scale Proteomics Studies,” Journal of Proteome Research 9, no. 8 (2010): 4152–4160, 10.1021/pr1003856. 20578722 PMC3221600

[18] C. Simon , K. Duehrkop , D. Petras , et al., “Mass Difference Matching Unfolds Hidden Molecular Structures of Dissolved Organic Matter,” Environmental Science & Technology 56 (2022): 11027–11040, 10.1021/acs.est.2c01332. 35834352 PMC9352317

[19] J. Kaeslin and R. Zenobi , “Resolving Isobaric Interferences in Direct Infusion Tandem Mass Spectrometry,” Rapid Communications in Mass Spectrometry 36, no. 9 (2022): e9266, 10.1002/rcm.9266. 35124854 PMC9286799

[20] M. A. Moseley , C. J. Hughes , P. R. Juvvadi , et al., “Scanning Quadrupole Data‐Independent Acquisition, Part A: Qualitative and Quantitative Characterization,” Journal of Proteome Research 17, no. 2 (2018): 770–779, 10.1021/acs.jproteome.7b00464. 28901143 PMC12140809

[21] C. B. Messner , V. Demichev , N. Bloomfield , et al., “Ultra‐Fast Proteomics With Scanning SWATH,” Nature Biotechnology 39, no. 7 (2021): 846–854, 10.1038/s41587-021-00860-4. PMC7611254 33767396

[22] T. Kind , H. Tsugawa , T. Cajka , et al., “Identification of Small Molecules Using Accurate Mass MS/MS Search,” Mass Spectrometry Reviews 37, no. 4 (2018): 513–532, 10.1002/mas.21535. 28436590 PMC8106966

[23] M. Mann and N. L. Kelleher , “Precision Proteomics: The Case for High Resolution and High Mass Accuracy,” Proceedings of the National Academy of Sciences of the United States of America 105, no. 47 (2008): 18132–18138, 10.1073/pnas.0800788105. 18818311 PMC2587563

[24] P. Stincone , A. P. Shah , R. Schmid , et al., “Evaluation of Data‐Dependent MS/MS Acquisition Parameters for Non‐Targeted Metabolomics and Molecular Networking of Environmental Samples: Focus on the Q Exactive Platform,” Analytical Chemistry 95, no. 34 (2023): 12673–12682, 10.1021/acs.analchem.3c01202. 37578818 PMC10469366

[25] A. Kalli and S. Hess , “Effect of Mass Spectrometric Parameters on Peptide and Protein Identification Rates for Shotgun Proteomic Experiments on an LTQ‐Orbitrap Mass Analyzer,” Proteomics 12, no. 1 (2012): 21–31, 10.1002/pmic.201100464. 22065615

[26] M. V. Gorshkov , D. M. Good , Y. Lyutvinskiy , H. Q. Yang , and R. A. Zubarev , “Calibration Function for the Orbitrap FTMS Accounting for the Space Charge Effect,” Journal of The American Society for Mass Spectrometry 21, no. 11 (2010): 1846–1851, 10.1016/j.jasms.2010.06.021. 20696596

[27] L. Sleno and D. A. Volmer , “Ion Activation Methods for Tandem Mass Spectrometry,” Journal of Mass Spectrometry 39, no. 10 (2004): 1091–1112, 10.1002/jms.703. 15481084

[28] J. L. Bushee and U. A. Argikar , “An Experimental Approach to Enhance Precursor Ion Fragmentation for Metabolite Identification Studies: Application of Dual Collision Cells in an Orbital Trap,” Rapid Communications in Mass Spectrometry 25, no. 10 (2011): 1356–1362, 10.1002/rcm.4996. 21504000

[29] R. Adusumilli and P. Mallick , “Data Conversion With ProteoWizard msConvert,” Methods in Molecular Biology 1550 (2017): 339–368, 10.1007/978-1-4939-6747-6_23 New York, NY, Springer New York. 28188540

[30] M. C. Chambers , B. Maclean , R. Burke , et al., “A Cross‐Platform Toolkit for Mass Spectrometry and Proteomics,” Nature Biotechnology 30, no. 10 (2012): 918–920, 10.1038/nbt.2377. PMC3471674 23051804

[31] H. Wickham , ggplot2: Elegant Graphics for Data Analysis (Springer‐Verlag New Yor, 2016).

[32] H. Wickham stringr: Simple, Consistent Wrappers for Common String Operations. Version 1.5.1 2023. https://stringr.tidyverse.org.

[33] Girlich HWaDVaM . Tidyr: Tidy Messy Data. Version 1.3.1. 2025. https://tidyr.tidyverse.org.

[34] H. W. Vaughan , aRFaLHaKMaD.Dplyr: A Grammar of Data Manipulation. Version 1.1.4. 2025. https://dplyr.tidyverse.org.

[35] J. Stanstrup , C. D. Broeckling , R. Helmus , et al., “The metaRbolomics Toolbox in Bioconductor and Beyond,” Metabolites 9, no. 10 (2019): 200, 10.3390/metabo9100200. 31548506 PMC6835268

[36] K. Duhrkop , M. Fleischauer , M. Ludwig , et al., “SIRIUS 4: a Rapid Tool for Turning Tandem Mass Spectra Into Metabolite Structure Information,” Nature Methods 16, no. 4 (2019): 299–302, 10.1038/s41592-019-0344-8. 30886413

[37] H. Yang , C. X. Yang , and T. L. Sun , “Characterization of Glycopeptides Using a Stepped Higher‐Energy C‐Trap Dissociation Approach on a Hybrid Quadrupole Orbitrap,” Rapid Communications in Mass Spectrometry 32, no. 16 (2018): 1353–1362, 10.1002/rcm.8191. 29873418

[38] J. K. Diedrich , A. F. M. Pinto , and J. R. Yates , “Energy Dependence of HCD on Peptide Fragmentation: Stepped Collisional Energy Finds the Sweet Spot,” Journal of The American Society for Mass Spectrometry 24, no. 11 (2013): 1690–1699, 10.1007/s13361-013-0709-7. 23963813 PMC3815594

[39] R. L. Cai , W. Huang , M. Meder , et al., “Improving the Sensitivity of Fourier Transform Mass Spectrometer (Orbitrap) for Online Measurements of Atmospheric Vapors,” Analytical Chemistry 94, no. 45 (2022): 15746–15753, 10.1021/acs.analchem.2c03403. 36342268 PMC9670027

[40] M. S. Kim , L. Zhou , M. Choi , Y. L. Zhang , Y. Q. Zhou , and K. S. Jang , “Molecular Characterization of Dissolved Organic Matter Unveils Their Complexity, Origin, and Fate in Glacier and Glacial‐Fed Streams and Lakes on the Tibetan Plateau,” Mass Spectrom Lett 12, no. 4 (2021): 192–199, 10.5478/Msl.2021.12.4.192.

[41] E. Niyonsaba , J. M. Manheim , R. Yerabolu , and H. I. Kenttämaa , “Recent Advances in Petroleum Analysis by Mass Spectrometry,” Analytical Chemistry 91, no. 1 (2019): 156–177, 10.1021/acs.analchem.8b05258. 30428670

