# Supplementary material for: Enhancing Chimeric Fragmentation Spectra Deconvolution Using Direct Infusion–Tandem Mass Spectrometry Across High‐Resolution Mass Spectrometric Platforms
Source: Rapid Commun Mass Spectrom. 2025 Nov 16;40(3):e10170. doi: 10.1002/rcm.10170 (PMC12620602; doi:10.1002/rcm.10170)
Supplement: Supplementary file 1 — Table S1: Properties of used compounds. Table S2: The detailed ESI source settings. Table S3: Number of peaks in each reconstructed pseudo‐individual MS2 spectrum compared with corresponding reference spectrum: in blue—correctly assigned peaks; in grey—missing peaks; in red—incorrectly assigned peaks that belong to the other isobar in isobaric mixture. Table S4: Length of one measurement cycle under different instrumental settings. Table S5: Shared fragmentation peaks of isobaric mixtures. Table S6: Estimated NCE50 (normalised collision energy at which the precursor ion accounts for 50% of the total MS2 signal intensity), predicted collision cross section* and width of modulated intensity profiles for three isobaric mixtures. Figure S1: Shapes of the modulated precursors' intensities observed for different isobaric mixtures measured at 35 NCE with otherwise default settings, Δm/z corresponds to the difference between m/z values of isobars' [M + H] + ions. The top row recorded with LIT‐Orbitrap and bottom row recorded with Q‐Orbitrap. While there is no clear connection between Δm/z values and the difference between isobars' modulated intensities for LIT‐Orbitrap, the bigger Δm/z generally lead to larger shift between modulated intensities for Q‐Orbitrap. Figure S2: Distance between modulated intensity profiles for isobaric pairs from Figure 3 and Figure S1 changing with m/z differences between isobars' [M + H] + ions (right). The distance between modulated intensities is calculated as a number of scans between the MS2 events, in which one of isobars reaches relative intensity of 0.5 (e.g., if precursor A reaches 0.5 relative intensity at scans 30 and 60 and precursor B—at scans 35 and 65, then the distance between modulated intensity profiles of isobars A and B is |35–30| + |65–60| = 10 scans; left). The data were measured at 35 NCE with otherwise default settings. While there is no clear connection between m/z differences and the distance between isobars' modulated [file RCM-40-e10170-s001.pdf]

Enhancing Chimeric Fragmentation Spectra Deconvolution using Direct Infusion – Tandem Mass Spectrometry across High-Resolution Mass Spectrometric Platforms

Arina Ivanova[1], Wei Tang[2], Carsten Simon[1§], Kai Dührkop[2], Sebastian Böcker[2] and Gerd Gleixner[1]

[1]Department of Biogeochemical Processes, Max Planck Institute for Biogeochemistry, Jena, Germany

[2]Chair for Bioinformatics, Friedrich Schiller University Jena, Jena, Germany

[§] Present address: Helmholtz Centre for Environmental Research-UFZ, Department of Environmental Analytical Chemistry, Leipzig, Germany

Corresponding author: Gerd Gleixner (gerd.gleixner@bgc-jena.mpg.de)

Supplementary materials

Table of Contents

Supplementary Table 1. Properties of used compounds **2**

Supplementary Table 2. The detailed ESI source settings **2**

Supplementary Table 3. Number of peaks in each reconstructed pseudo-individual MS2 spectrum compared to corresponding reference spectrum........ **3**

Supplementary Table 4. Length of one measurement cycle under different instrumental settings **4**

Supplementary Table 5. Shared fragmentation peaks of isobaric mixtures........ **4**

Supplementary Table 6. Estimated NCE50, predicted collision cross-section and width of modulated intensity profiles........ **5**

Supplementary Figure 1. Shapes of the modulated precursors' intensities observed for different isobaric mixtures. **5**

Supplementary Figure 2. Distance between modulated intensity profiles for isobaric pairs.. **6**

Supplementary Figure 3. Recall of reconstructed individual fragmentation spectra obtained under different instrumental settings. **7**

Supplementary Figure 4. Average mass error before recalibration at different AGC values. **8**

Supplementary Figure 5. Average correlation value between fragments and precursors obtained under different instrumental settings on LIT-Orbitrap and Q-Orbitrap. **8**

Supplementary Figure 6. Comparison of intensity modulation profiles of precursors and their most intense fragments when using different isolation window width. **9**

Supplementary Figure 7. The sections of MS1 spectra of isobaric mixture 342A+342B. **9**

Supplementary Figure 8. Modulated precursors' intensity profiles observed for the same isobaric mixture at different concentrations. **10**

Supplementary Table 1. Properties of used compounds

Molecular formula	Mono-isotopic mass (neutral)	*m/z* of $[M+H]^+$	ID	IUPAC name	InChIKey	Canonical SMILES	Structure
$C_5H_{12}N_2O_3S$	180.0569	181.0641	180E	2-amino-4-(methylsulfonimidoyl)butanoic acid	SXTAYKAGBXMACB-DPVSGNNYSA-N	CS(=N)(=O)CCC(C(=O)O)N	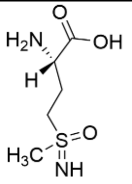
$C_{10}H_{12}O_3$	180.0786	181.0859	180G	ethyl 2-hydroxy-6-methylbenzoate	KWXBNUYCDMPLEQ-UHFFFAOYSA-N	CCOC(=O)C1=C(C=CC=C1O)C	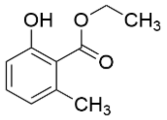
$C_{17}H_{14}N_2O_4S$	342.0674	343.0747	342A	N'-[(Z)-(2,3-dihydroxyphenyl)methylidene]naphthalene-2-sulfonohydrazide	JHBRHEKBSGQYKF-WQRHYEAKSA-N	C1=CC=C2C=C(C=CC2=C1)S(=O)(=O)NN=CC3=C(C(=CC=C3)O)O	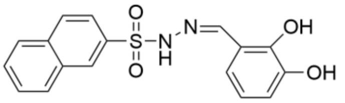
$C_{18}H_{14}O_7$	342.0739	343.0812	342B	{[3-(4-Methoxyphenoxy)-4-oxo-4H-chromen-7-yl]oxy}acetic acid	XLYKDLBSXBWYKC-UHFFFAOYSA-N	COC1=CC=C(C=C1)OC2=COC3=C(C2=O)C=CC(=C3)OCC(=O)O	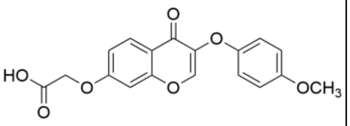
$C_{18}H_{18}N_2O_5$	342.1216	343.1288	342F	2-[4-[(E)-[[2-(3-methylphenoxy)acetyl]hydrazinylidene]methyl]phenoxy]acetic acid	MGIRZVBXORKNAX-VXLYETTFSA-N	CC1=CC(=CC=C1)OCC(=O)NN=CC2=CC=C(C=C2)OCC(=O)O	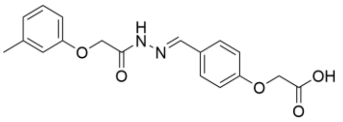

Supplementary Table 2. The detailed ESI source settings

	Orbitrap Elite (LIT-Orbitrap)	Orbitrap Exploris 120 (Q-Orbitrap)
**Flow rate, µl/min**	20	5
**Capillary voltage, kV**	4	4
**Sheath gas, arb**	10	5
**Auxiliary gas, arb**	5	3
**Sweep gas, arb**	5	1
**Capillary T, °C**	275	275
**S lens RF level**	69	70

Supplementary Table 3. Number of peaks in each reconstructed pseudo-individual MS2 spectrum compared to corresponding reference spectrum: in blue – correctly assigned peaks; in grey – missing peaks; in red – incorrectly assigned peaks that belong to the other isobar in isobaric mixture.

	**Number of peaks assigned to the reconstructed pseudo-individual fragmentation spectrum**											
	**LIT-Orbitrap**						**Q-Orbitrap**					
	180E+180G		342A+342B		342B+342F		180E+180G		342A+342B		342B+342F	
	180E	180G	342A	342B	342B	342F	180E	180G	342A	342B	342B	342F
**Default**	3<br>4<br>0	7<br>0<br>0	20<br>4<br>0	14<br>8<br>0	15<br>7<br>0	36<br>17<br>0	11<br>0<br>0	3<br>2<br>0	1<br>24<br>0	20<br>8<br>19	17<br>10<br>0	39<br>10<br>0
**0.01 *m/z* step size**	3<br>4<br>0	7<br>0<br>0	18<br>6<br>0	17<br>5<br>0	16<br>6<br>0	36<br>17<br>0	11<br>0<br>0	3<br>2<br>0	1<br>24<br>0	21<br>6<br>18	18<br>9<br>0	39<br>10<br>0
**0.04 *m/z* step size**	3<br>4<br>0	7<br>0<br>0	21<br>3<br>0	15<br>7<br>0	15<br>7<br>0	37<br>16<br>0	10<br>1<br>0	5<br>0<br>0	1<br>24<br>0	20<br>7<br>19	19<br>8<br>0	37<br>12<br>0
**0.1 *m/z* step size**	2<br>5<br>0	7<br>0<br>0	20<br>4<br>0	15<br>7<br>0	16<br>6<br>0	36<br>17<br>0	10<br>1<br>1	4<br>1<br>0	1<br>24<br>0	20<br>7<br>19	20<br>7<br>1	36<br>13<br>0
**1 microscan**	3<br>4<br>0	7<br>0<br>0	22<br>2<br>0	15<br>7<br>0	15<br>7<br>0	37<br>16<br>0	9<br>2<br>0	5<br>0<br>0	1<br>24<br>0	21<br>6<br>20	19<br>8<br>4	38<br>11<br>0
**3 microscans**	3<br>4<br>0	7<br>0<br>0	21<br>3<br>0	15<br>7<br>0	15<br>7<br>0	36<br>17<br>0	10<br>1<br>0	5<br>0<br>0	1<br>24<br>0	21<br>7<br>17	17<br>10<br>0	39<br>10<br>0
**R = 15 000**	3<br>4<br>0	7<br>0<br>0	22<br>2<br>0	16<br>6<br>0	17<br>5<br>0	36<br>17<br>0	1<br>10<br>0	5<br>0<br>9	21<br>4<br>5	14<br>13<br>0	20<br>6<br>29	2<br>48<br>1
**R = 30 000**	3<br>4<br>0	7<br>0<br>0	22<br>2<br>0	18<br>4<br>0	17<br>5<br>0	36<br>17<br>0	10<br>1<br>0	4<br>1<br>1	20<br>5<br>4	16<br>11<br>0	20<br>7<br>22	13<br>36<br>1
**R = 60 000**	3<br>4<br>0	7<br>0<br>0	22<br>2<br>0	16<br>6<br>0	16<br>6<br>0	37<br>16<br>0	10<br>1<br>0	4<br>1<br>1	18<br>7<br>6	38<br>11<br>2	14<br>13<br>1	38<br>11<br>2
**0.7 m/z isolation width**	2<br>5<br>0	7<br>0<br>0	19<br>5<br>0	16<br>6<br>0	15<br>7<br>0	35<br>18<br>0	10<br>1<br>0	5<br>0<br>0	1<br>24<br>0	20<br>7<br>19	19<br>8<br>0	38<br>11<br>0
**2 m/z isolation width**	5<br>2<br>0	7<br>0<br>0	20<br>4<br>0	14<br>8<br>0	14<br>8<br>0	36<br>17<br>0	3<br>8<br>0	4<br>1<br>7	1<br>24<br>0	21<br>6<br>18	17<br>10<br>0	38<br>11<br>1
**50% AGC**	2<br>5<br>0	4<br>3<br>0	20<br>4<br>0	15<br>7<br>0	14<br>8<br>0	38<br>15<br>1	10<br>1<br>0	5<br>0<br>0	1<br>24<br>0	21<br>6<br>19	19<br>8<br>0	37<br>12<br>0
**200% AGC**	3<br>4<br>0	7<br>0<br>0	20<br>4<br>0	15<br>7<br>0	14<br>8<br>0	37<br>16<br>0	10<br>1<br>0	5<br>0<br>0	1<br>24<br>0	21<br>6<br>17	17<br>10<br>0	39<br>10<br>0
**NCE = 35**	3<br>4<br>0	3<br>0<br>0	18<br>3<br>0	13<br>1<br>0	10<br>4<br>0	25<br>12<br>0	8<br>3<br>0	3<br>0<br>3	1<br>20<br>0	15<br>3<br>16	14<br>4<br>0	25<br>9<br>0
**NCE = 50**	4<br>4<br>0	4<br>1<br>0	22<br>3<br>0	18<br>14<br>0	19<br>13<br>0	34<br>18<br>0	8<br>3<br>0	3<br>1<br>1	20<br>6<br>12	9<br>21<br>2	16<br>14<br>0	40<br>10<br>2
**NCE = 65**	4<br>2<br>0	6<br>0<br>1	23<br>3<br>0	17<br>18<br>1	11<br>24<br>0	49<br>18<br>1	8<br>0<br>0	3<br>2<br>0	22<br>9<br>2	23<br>7<br>0	16<br>14<br>0	46<br>12<br>0

Supplementary Table 4. Length of one measurement cycle under different instrumental settings

**Settings**	**Length of one measurement cycle, s**							
	**LIT-Orbitrap**				**Q-Orbitrap**			
	180E+180G	342A+342B	342B+342F	Average ± SD*	180E+180G	342A+342B	342B+342F	Average ± SD*
**Default**	422	380	376	393±25	105.0	104.7	104.7	104.8±0.2
**0.01 step**	850	766	771	796±47	211.0	211.0	211.0	211.0±0.0
**0.04 step**	203	182	182	189±12	52.4	52.4	52.4	52.4±0.0
**0.1 step**	81	73	73	76±5	23.7	20.9	21.3	22.0±1.5
**1 microscan**	136	119	121	125±9	20.9	20.9	20.9	20.9±0.0
**3 microscans**	272	239	244	251±18	62.8	62.8	62.8	62.8±0.0
**R = 15 000**	265	208	240	237±29	7.3	14.7	14.7	12.2±4.2
**R = 30 000**	278	225	257	253±27	27.5	27.5	27.5	27.5±0.0
**R = 60 000**	318	269	297	295±24	53.2	53.2	53.2	53.2±0.0
**0.7 *m/z* isolation width**	448	408	409	421±23	105.0	104.7	104.7	104.8±0.2
**2 *m/z* isolation width**	602	573	607	594±18	178.2	178.3	178.2	178.2±0.1
**50% AGC**	458	345	361	388±61	104.7	104.7	104.7	104.7±0.0
**200% AGC**	433	374	397	401±30	104.7	104.7	104.7	104.7±0.0
**NCE = 35**	260	265	254	260±5	104.7	104.7	104.7	104.7±0.0
**NCE = 50**	260	243	234	246±13	104.7	104.7	104.7	104.7±0.0
**NCE = 65**	260	265	254	260±5	104.7	104.7	104.7	104.7±0.0

*SD – one standard deviation

Supplementary Table 5. Shared fragmentation peaks of isobaric mixtures

**Isobaric pair**	**Collision energy**	**Number of fragments shared by isobars at given collision energy**		**Ratio of total peak intensity that corresponds to shared fragments, as per reference fragmentation spectra**	
		**On Q-Orbitrap**	**On LIT-Orbitrap**	**On Q-Orbitrap**	**On LIT-Orbitrap**
**180E+180G**	35	0	0	0	0
	50	0	0	0	0
	65	0	0	0	0
	35-45-55	0	0	0	0
**342A+342B**	35	2	1	342A: 0.17<br>342B: 0.12	342A: 0.14<br>342B: <0.005
	50	3	2	342A: 0.25<br>342B: 0.49	342A: 0.23<br>342B: 0.02
	65	6	2	342A: 0.26<br>342B: 0.49	342A: 0.22<br>342B: 0.03
	35-45-55	3	2	342A: 0.22<br>342B: 0.33	342A: 0.18<br>342B: 0.01
**342B+342F**	35	3	3	342B: <0.005<br>342F: 0.11	342B: <0.005<br>342F: 0.13
	50	6	7	342B: 0.08<br>342F: 0.05	342B: 0.08<br>342F: 0.13
	65	7	8	342B: 0.24<br>342F: 0.13	342B: 0.28<br>342F: 0.14
	35-45-55	6	6	342B: 0.05<br>342F: 0.13	342B: 0.06<br>342F: 0.11

Supplementary Table 6. Estimated NCE50 (normalized collision energy at which the precursor ion accounts for 50% of the total MS2 signal intensity**),** predicted collision cross-section* and width of modulated intensity profiles for three isobaric mixtures

**Pair of isobars**	**Molecular formula**	**Estimated NCE50**	**Predicted collision cross-section, Å**	**Width of intensity profile at 50% of normalized intensity, scans**
318A + 318F	318A: $C_{14}H_{10}N_2O_5S$	318A: 25	172.1	318A: 52
	318F: $C_{16}H_{18}N_2O_5$	318F: 22	171.9	318F: 31
302B + 302D	302B: $C_{15}H_{10}O_7$	302B: 52	165.0	302B: 61
	302D: $C_{16}H_{14}O_6$	302D: 28	165.6	302D: 45
342B + 342F	342B: $C_{18}H_{14}O_7$	342B: 41	176.0	342B: 52
	342F: $C_{18}H_{18}N_2O_5$	342F: 25	182.4	342F: 42

*collision cross-section was predicted for $[M+H]^+$ precursor ions via https://ccsbase.net/predictions

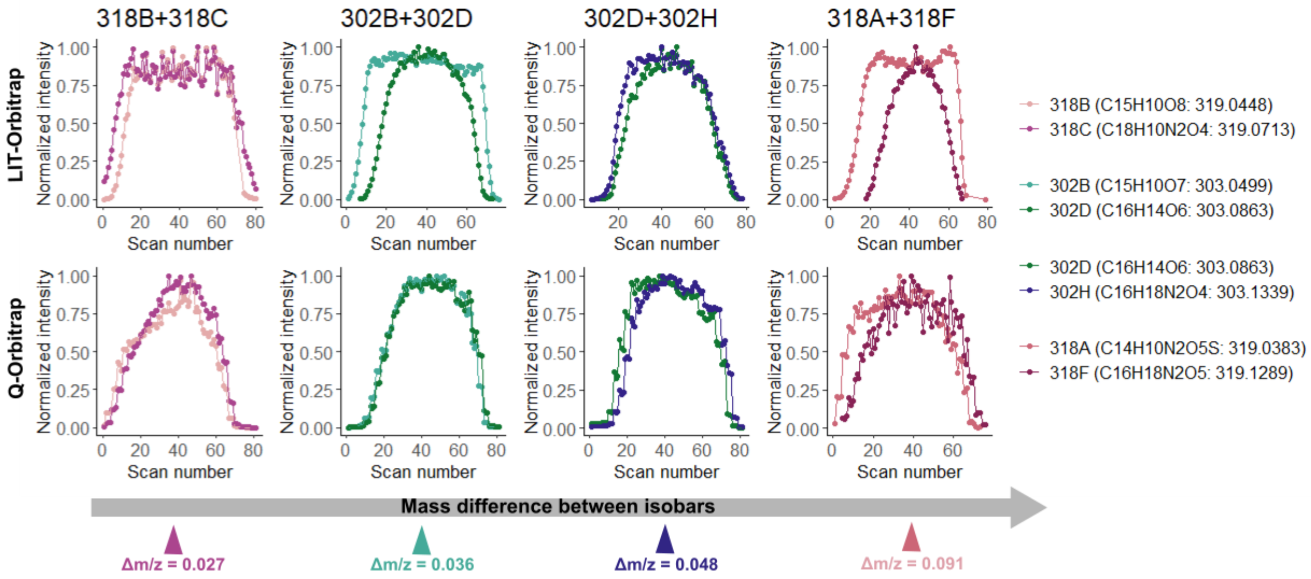

Supplementary Figure 1. Shapes of the modulated precursors' intensities observed for different isobaric mixtures measured at 35 NCE with otherwise default settings, *Δm/z* corresponds to the difference between *m/z* values of isobars' $[M+H]^+$ ions. The top row recorded with LIT-Orbitrap and bottom row recorded with Q-Orbitrap. While there is no clear connection between *Δm/z* values and the difference between isobars' modulated intensities for LIT-Orbitrap, the bigger *Δm/z* generally lead to larger shift between modulated intensities for Q-Orbirap.

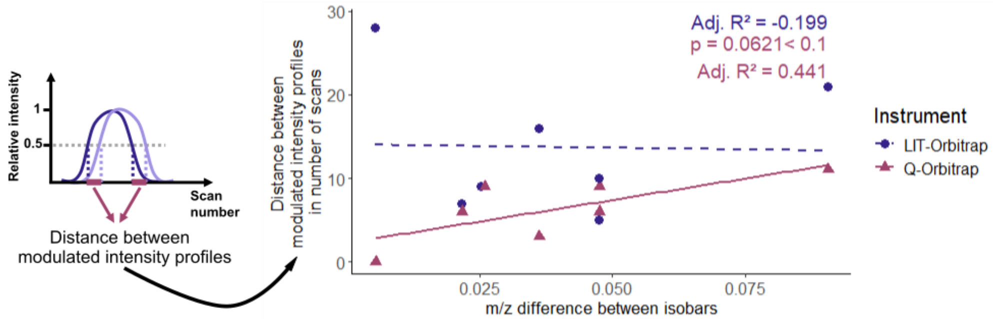

Supplementary Figure 2. Distance between modulated intensity profiles for isobaric pairs from Figure 3 and Supplementary Figure 1 changing with *m/z* differences between isobars' $[M+H]^+$ ions (right). The distance between modulated intensities is calculated as a number of scans between the MS2 events, in which one of isobars reaches relative intensity of 0.5 (e.g., if precursor A reaches 0.5 relative intensity at scans 30 and 60 and precursor B – at scans 35 and 65, then the distance between modulated intensity profiles of isobars A and B is |35-30| + |65-60| = 10 scans; left). The data was measured at 35 NCE with otherwise default settings. While there is no clear connection between *m/z* differences and the distance between isobars' modulated intensities for LIT-Orbitrap, there is a weak trend for Q-Orbitrap: bigger *m/z* differences lead to larger shift between modulated intensities.

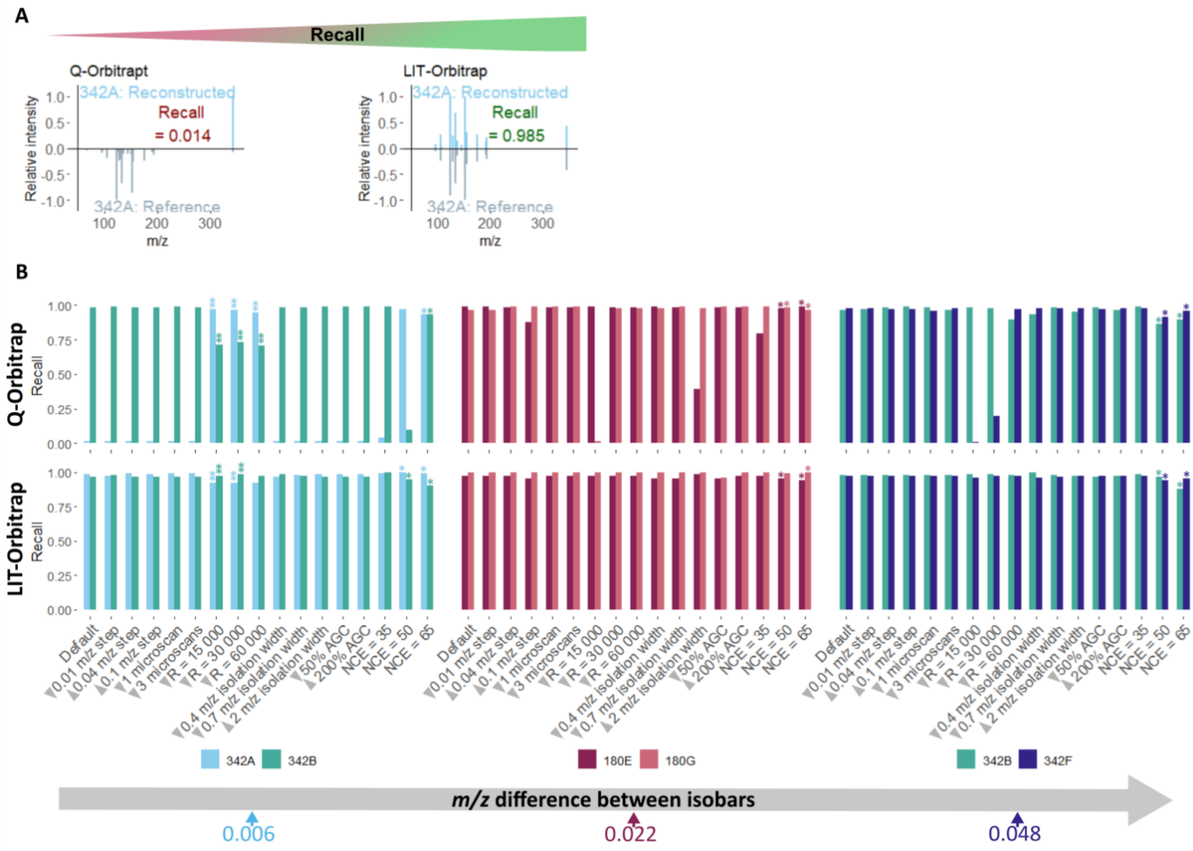

Supplementary Figure 3. A) Example of mirror plots comparing an isobar's reconstructed individual MS2 spectrum and the respective reference spectrum (here, compound 342A) with corresponding recall. (0 – no peaks present in the reference spectrum were assigned to a reconstructed spectrum, i.e. no peaks were correctly assigned; 1 – all peaks present in reference spectrum were assigned to a reconstructed spectrum). Data was recorded with default settings. B) Recall of reconstructed individual fragmentation spectra obtained under different instrumental settings on Q-Orbitrap (upper row) and LIT-Orbitrap (lower row). Different colours denote different isobaric compounds. * isobar is fully fragmented at this collision energy, the most intense fragment is used instead of precursor in correlation analysis; ** mass resolution is too low to separate isobaric peaks, the most intense fragment is used instead of precursor in correlation analysis. Δ – the value of the setting is higher than in the default method; ∇ –the value is lower.

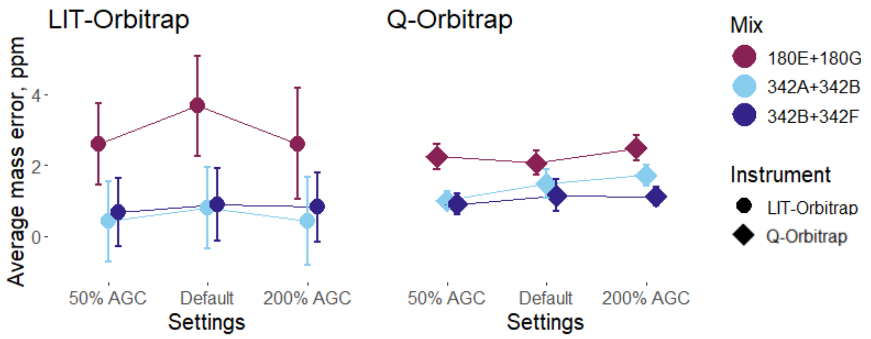

Supplementary Figure 4. Average mass error before recalibration at different AGC values for three tested isobaric mixtures and two mass spectrometers. Only measurements performed at the same day are considered to exclude day-to-day variation in mass accuracy. Error bars are given at one standard deviation level.

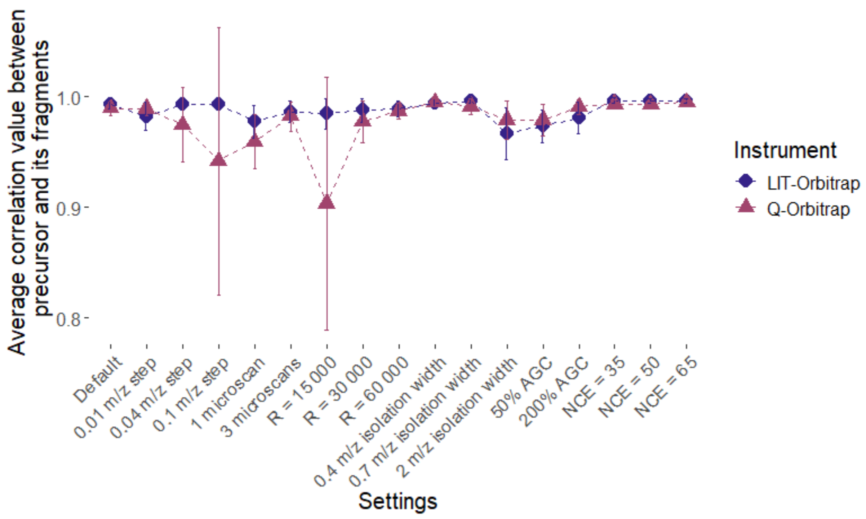

Supplementary Figure 5. Average correlation value between fragments and precursors obtained under different instrumental settings on LIT-Orbitrap and Q-Orbitrap. The correlation value for each setting is averaged over all six isobars from three isobaric mixtures.

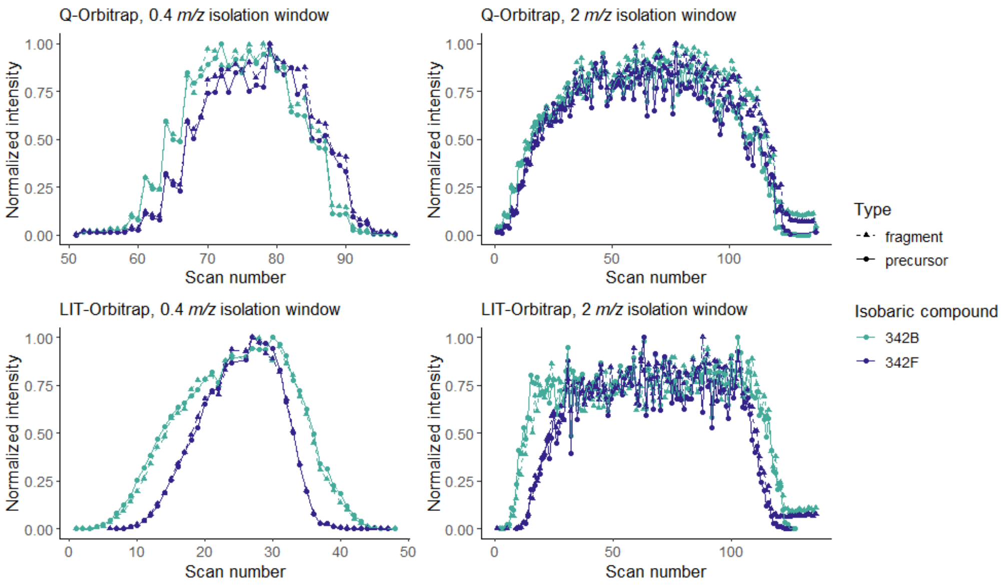

Supplementary Figure 6. Comparison of intensity modulation profiles of precursors and their most intense fragments when using different isolation window width.

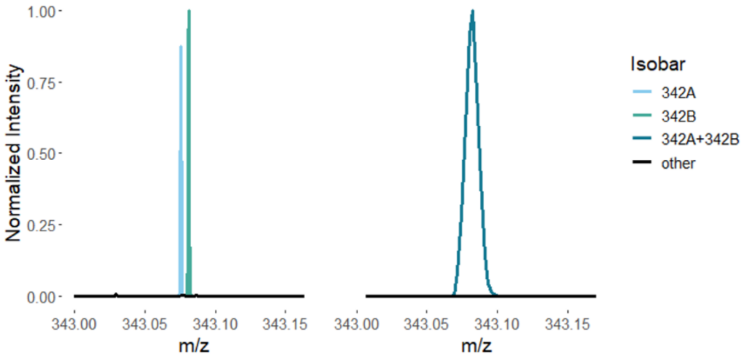

Supplementary Figure 7. The sections of MS1 spectra of isobaric mixture 342A+342B recorded at mass resolution 480 000 (left) and 30 000 (right) on LIT-Orbitrap. The individual isobaric peaks cannot be separated at mass resolution below 60 000 for LIT-Orbitrap and 120 000 for Q-Orbitrap.

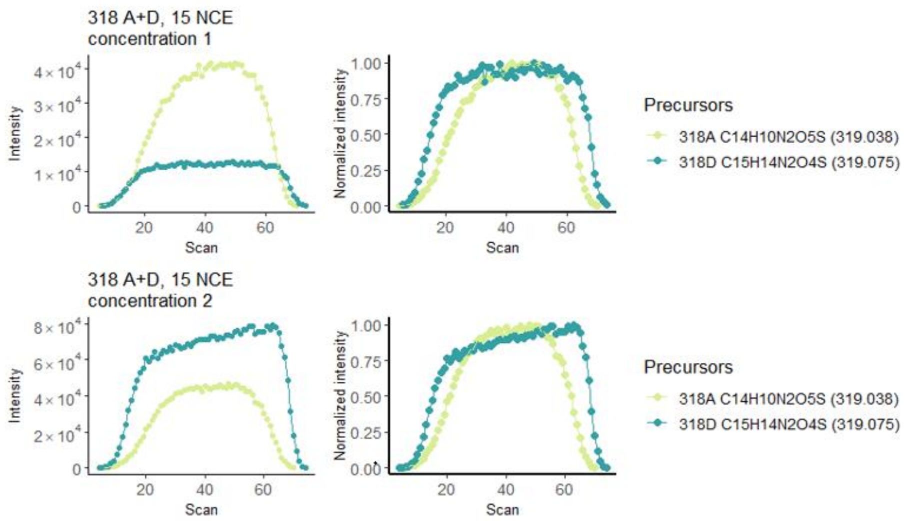

Supplementary Figure 8. Modulated precursors' intensity profiles observed for the same isobaric mixture at different concentrations. Compound 318D has "wider" modulated intensity profile also when its intensity is lower than intensity of compound 318A (upper row).
